# LPS^low^-Macrophages Alleviate the Outcome of Graft-*Versus*-Host Disease Without Aggravating Lymphoma Growth in Mice

**DOI:** 10.3389/fimmu.2021.670776

**Published:** 2021-08-03

**Authors:** Mohamed Jeljeli, Charlotte Chêne, Sandrine Chouzenoux, Marine Thomas, Benjamin Segain, Ludivine Doridot, Carole Nicco, Frédéric Batteux

**Affiliations:** ^1^Département 3I «Infection, Immunité et Inflammation», Institut Cochin, INSERM U1016, Université de Paris, Paris, France; ^2^Université de Paris, Faculté de Médecine, AP-HP-Centre Université de Paris, Hôpital Cochin, Service d’immunologie biologique, Paris, France

**Keywords:** macrophages, trained immunity, graft-*versus*-host disease, alloimmunity, inflammation, anti-tumoral action

## Abstract

Despite significant therapeutic advances, graft-*versus*-host disease (GvHD) remains the main life-threatening complication following allogeneic hematopoietic stem cell transplantation. The pathogenesis of GvHD is dominated by a dysregulated allogeneic immune response that drives fibrosis and autoimmunity in chronic forms. A multitude of cell therapy approaches, including infusion of myeloid cells, has been proposed to prevent GvHD through tolerance induction but yielded variable results. Myeloid cells like macrophages can be reprogrammed to develop adaptive-like features following antigenic challenge to reinforce or inhibit a subsequent immune response; a phenomenon termed ‘trained immunity’. Here we report that, whereas LPS^low^-trained macrophages elicit a suppressor effect on allogeneic T cell proliferation and function *in vitro* in an IL-10-dependent manner, Bacille Calmette et Guérin (BCG)-trained macrophages exert an opposite effect. In a murine model of sclerodermatous chronic GvHD, LPS^low^-trained macrophages attenuate clinical signs of GvHD with significant effects on T cell phenotype and function, autoantibodies production, and tissue fibrosis. Furthermore, infusion of LPS^low^-macrophages significantly improves survival in mice with acute GvHD. Importantly, we also provide evidence that LPS^low^-macrophages do not accelerate A20-lymphoma tumor growth, which is significantly reduced upon transfer of BCG-macrophages. Collectively, these data indicate that macrophages can be trained to significantly inhibit *in vitro* and *in vivo* allo-reactive T cell proliferation without exhibiting pro-tumoral effect, thereby opening the way to promising clinical applications.

## Introduction

Graft-*versus*-host disease (GvHD) constitutes the leading cause of morbidity and non-relapse related mortality after allogeneic hematopoietic stem cell transplantation (HSCT) (1, 2). From the standpoint of hematological malignancies, GvHD treatment remains challenging as allo-reactive T cell depletion by aggressive immunosuppressive therapies can dampen the graft-*versus*-leukemia effect with a significantly increased risk of relapse of the primary malignancy (3, 4). Despite the dualistic classification of GvHD into acute and chronic forms, based on a cutoff of 100 days of clinical manifestation(s) following HSCT, involvement of the skin and the gastro-intestinal (GI) tract is the constitutive hallmark of these disorders (5). Of note, a patho-physiological continuum exists between the acute and chronic forms of GvHD with inflammation as a trigger for the dysregulated immune response against host tissues (6). Indeed, inflammatory processes play a key role in the initiation and maintenance of immunological mechanisms that underlie alloantigen recognition of host antigens and subsequent tissue destruction by effector T cells (7, 8). The cellular damage induced by conditioning regimen leads to the massive release of damage-associated molecular patterns (DAMPs) and pathogen-associated molecular patterns (PAMPs) triggered by the leakage of gut bacteria and activation of tissue dendritic cells and macrophages (6). The subsequent production of pro-inflammatory cytokines such as Interleukin (IL)-6, IL-1β and Interferon (IFN)-*α* favors the differentiation of donor CD4^+^ T cells into Th1 and Th17 inflammatory effector subsets (9). This balance towards a hyperinflammatory status, triggered by the innate immune response, increases the activation of the donor allogeneic CD4^+^ T cells and enhances their recognition of host allo-antigens presented by activated dendritic cells and macrophages (7). Recently, an emerging concept termed ‘trained immunity’ has been demonstrated whereby innate immune cells like macrophages and NK cells display adaptive features with a rudimentary memory to past antigenic challenges (10–12). Numerous studies conducted by Netea and colleagues have demonstrated that macrophages exposed to BCG or *β*-Glucan from *Candida albicans* show a heightened capacity to produce pro-inflammatory cytokines and an upregulation of activation markers and recognition receptors following re-stimulation with pathogens or inflammatory stimuli (13–15). In contrast, macrophages can also acquire a hypo-responsive memory and mount an immunosuppressive response upon exposure to certain stimuli such as helminth products or chronic stimulation with bacterial endotoxin (LPS) (16–19). Trained immunity is accompanied by profound changes in cellular metabolic pathways that regulate chromatin-modifying enzymes leading to long lasting epigenetic modifications (20). Studies targeting trained immunity have mostly focused on the enhanced activation of the immune cells and inflammation to target impaired immune responses and to improve vaccination scheme (21, 22). We recently reported that macrophages trained with low dose of LPS (LPS^low^-macrophages) were able to reduce inflammation-induced fibrosis in a murine model of systemic sclerosis, while BCG-trained macrophages (BCG-macrophages) worsened it (23). Clinical strategies to reduce GvHD have focused on the use of immunosuppressive agents that non-specifically affect T-cell function and may be responsible for compromising T-cell immunity by reducing absolute T-cell numbers or inhibiting the function of existing donor T cells (24). Based on these observations, we assessed the effects of trained macrophages, especially the LPS^low^-trained macrophages on the allogeneic activity of T cells on the development of acute and chronic GvHD in a murine model. In parallel, we sought to analyze whether the effects observed could influence tumor growth/progression in the A20 B lymphoma murine model as a proof-of-concept for potential cellular therapeutic strategy.

## Materials and Methods

### Reagents and Chemicals

All chemicals were from Sigma Aldrich (Saint Quentin Fallavier, France). LPS was from *E. coli* serotype 0127: B8 (Sigma Aldrich); BCG vaccine was purchased from Sanofi Pasteur and composed of 0.5 mg of the Brazilian strain (BCG Biomed-Lublin Laboratory).

### Mice

For graft-*versus*-host disease experiments, eight-week-old female *Balb/c* (H2^d^) and C57BL6 (H2^b^), weighing 15–17 g, were purchased from Janvier Laboratory (Le Genest Saint Isle, France). Male *B10.D2* (H2^d^) mice were kindly offered by Colette Kanellopoulos-Langevin, CDTA-CNRS-Orléans, France). For the A20 lymphoma induction, eight-week-old *Balb/c* AnN strain mice (H2d) weighing 15–17 g were purchased from Janvier Laboratory (Le Genest Saint Isle, France). All mice were housed in ventilated cages with free access to food and water and maintained under standard 12-h photoperiod. Animals were treated humanely in compliance with the institutional guidelines. The protocols and all experimental procedures of this study were approved by the Ethics Committee from Paris Descartes University (N° CEEA34.CN.017.12).

### CD4^+^ T-Cell Purification and CFSE Labeling

The purification of CD4^+^ T cells derived from *B10.D2* mice (set as responder cells) was performed using the magnetic MACS separator from Miltenyi (130-110-443, Bergisch Gladbach, Germany) and compbeads CD4^+^ (01-1111-42, Invitrogen) for positive cell selection. Purity of the CD4^+^ T cell suspension obtained was verified by flow cytometry and was typically >95%. Splenic cells from *Balb/c* mice extracted as described above were irradiated at an intensity of 30 Gy to render them non-proliferative and constitute the stimulator cells. For CFSE labeling, irradiated splenocytes and CD4^+^ T cells were stained with 5 µM of CFSE (Ref C34554, Invitrogen) and incubated for 15 min at 37°C in a CO_2_ incubator protected from light. Labeling was stopped by adding complete MLR medium [RPMI with 10% FBS, 1% HEPES, 1% antibiotic and antimycotic (Gibco)].

### Bone Marrow Derived Macrophage Isolation and Innate Immune Training

Bone marrow (BM) cells were collected from the femurs and tibias of female *Balb/c* mice and differentiated into BMDM using BMDM medium (RPMI with 20% L929 cell supernatant, 10% FBS, 1% antibiotic/antimycotic, and 1% HEPES), as previously described (Manzanero, Methods Mol. Biol 2012; Weischenfeldt, CSH Protoc. 2008). At day 7, cells were washed in warm Ca^2+^/Mg^2+^-free PBS and collected by brief trypsin-EDTA (Ref 25200-056, Gibco) treatment. Cells were spun at 1,200 rpm for 7 min to form a pellet and then resuspended in complete RPMI medium. *In vitro* immune training of BMDMs was performed as previously described (23, 25). BCG treatment was achieved by 48 h incubation of macrophages with dry BCG vaccine (BCG Biomed-Lublin Laboratory) diluted in sterile PBS (Ref CS1PBS01-01, Eurobio) at a ratio of one bacillus for one cell followed by a resting phase of 3 days. LPS^low^ macrophage training was obtained by adding one daily dose of 10 ng/ml of LPS from *E. coli* serotype 0127:B8 (Sigma Aldrich) from day 1 to day 3 with daily change of culture medium to avoid cumulative dose toxicity. Control macrophages were obtained by daily treatment with 100 µl of sterile PBS. All cells were cultured in complete DMEM (Ref D0819-500, Sigma Aldrich) with 10% FCS and incubated at 37°C in an atmosphere containing 5% CO_2_. At day 7, cells were collected by brief trypsin-EDTA treatment, counted and used for either the MLR or for intraperitoneal injection of mice.

### Mixed Lymphocyte Reaction

Both the responder and the stimulator cells were adjusted at a density of 10^5^ per 200 µl of complete MLR medium and seeded in U-bottom 96-well microtiter plates (Ref 353077, Falcon). PBS, BCG, or LPS^low^-trained macrophages were obtained from *Balb/c* mice as described above and added at a concentration of 10^5^ to the respective wells as indicated. The assay included appropriate negative controls (CD4^+^ purified T cells and irradiated *Balb/c* splenocytes seeded alone) and positive control cultures (CD4^+^ T cells stimulated with 5 µg/ml of anti-CD3 (Ref 14-0032-86, eBioscience) and 2 µg/ml anti-CD28 (Ref 14-0281-86, eBioscience). Each condition of co-culture was performed in 10-plicate, and plates were incubated for 4 days at 37°C in 5% CO_2_.

### Macrophages IL-10 Invalidation With Small Interfering RNA

Differentiated bone marrow derived macrophages obtained from *Balb/c* mice were plated at 2 × 10^5^ cells per well in a six-well plate in 2 ml of antibiotic free normal growth medium supplemented with 10% of FBS (Gibco) for 24 h. The siRNA IL-10 (sc-39635) and control (scrambled sequence) siRNA (sc-37007) obtained from Santa Cruz Biotechnology were used to obtain IL-10 KO macrophages by using siRNA transfection medium (sc-36868) and siRNA transfection reagent (sc-29528) (Santa Cruz Biotechnology) according to the instructions of the manufacturer. Macrophages IL-10 invalidation was evaluated by Western blotting and ELISA measurement of IL-10 in stimulated LPS^low^-trained IL-10 KO macrophages.

### *In Vitro* Stimulation With Mouse Recombinant IL-10 and STAT3 Inhibition

The specific STAT3 Inhibitor V, Stattic sc-202818 (Santa Cruz Biotechnology, Catalog # 19983-44-9), was used as described before (26). A working solution of 500 µM was obtained after DMSO reconstitution and a 1/100 dilution in PBS. The CD4^+^ T cells were treated with 10 µM of the STAT3 Inhibitor V in 2 ml of complete RPMI medium (Ref R8758-500, Sigma Aldrich) supplemented with 10% heat inactivated Fetal Bovine Serum (FBS, Ref 10270-106, Gibco), 2 mM L-glutamine, 1% streptomycin–penicillin (Ref 15140-122, Gibco), 1 mM sodium pyruvate (Ref CSTVAT00-0U, Eurobio) and 10 mM HEPES (Ref 15630-056, Gibco). A control vehicle condition was composed of allogeneic CD4^+^ cells cultured in complete DMEM medium with PBS/DMSO (final concentration of 1% DMSO). Then, allogeneic CD4^+^ T cells were stimulated with the mouse recombinant IL-10 from BioLegend (Ref #575802, Ozyme France, 78180 Montigny-le-Bretonneux) at a concentration of 100 ng/ml and incubated for 24 h at 37°C 5% CO_2_. All the conditions were performed in six-plicate and the experiments were carried out twice. Cells were then collected to perform further analysis (Western blot and RT-qPCR).

### Clinical Assessment of GvHD

The severity of systemic GvHD in mice was assessed according to a mouse clinical GvHD scoring system inspired by Anderson et al. (27). We set the scoring system for chronic GvHD to five clinical criteria (maximum index = 5) representing the sum of the recorded values. Weight loss of <10% was scored 0 and of >20% was scored as 1. Regarding the GI symptoms, the scores were 0 for mice with no diarrhea and 1 for mice suffering from diarrhea. For posture, the scoring system denoted 0 for normal, 0.5 for hunching at rest, and 1 for severe hunching. For activity, the scoring represented 0 for normal, 0.5 for mild to moderate decrease, and 1 for severe decrease activity. For signs of vasculitis and cutaneous involvement, the scoring system denoted 0 for normal, 0.5 for mild lesions on ears or tails, and 1 for eyelid and severe alopecia. Total clinical GvHD score of each mouse was measured twice a week by a scientist blinded to the different groups.

### Isolation and Stimulation of Spleen Cells, Splenic Macrophages, and Skin-Derived Immune Cells

Mice were sacrificed by cervical dislocation. Spleens were aseptically removed, and cellular splenic suspensions were prepared after hypotonic lysis of erythrocytes in potassium acetate solution and three washes in complete RPMI medium supplemented with 10% heat inactivated Fetal Bovine Serum, 2 mM L-glutamine, 1% streptomycin–penicillin, 1 mM sodium pyruvate, and 10 mM HEPES. For each mouse, splenocytes were enumerated using a Malassez counting chamber. Skin-derived immune cell isolation was achieved as described before (28). Briefly, 8 mm-calibrated dermal punches of tissue were collected from the shaved back of each mouse and minced into small pieces in a 60 mm Petri dish. Tissue was then lysed with 1 ml of Liver Digest Medium (Gibco) solution and 1 mg/ml of collagenase (Ref C2674-1G, Sigma-Aldrich) in 9 ml of complete RPMI medium for 3 h at 37°C in an atmosphere of 5% CO_2_. Cell suspension was then filtered through a 70 µm cell strainer, washed in complete RPMI medium and counted with Malassez counting chamber for further analysis.

### Induction of cGvHD in *Balb/c* Mice and Trained Macrophage Infusion

Sclerodermatous chronic GvHD (cGvHD) was induced in female *Balb/c* mice (H^2d^) by grafting cells from 9- to 10-week-old male *B10.D2* mice (H^2d^), as previously described (29). Briefly, *Balb/c* mice were lethally irradiated with 750 cGy from a Gammacel [137Cs] source. Graft was prepared by adding 2 × 10^6^ spleen cells previously purified from red cells with a hypotonic solution of potassium acetate to 1 × 10^6^ of bone marrow cells resuspended in sterile PBS. Graft was injected in the retro-orbital vein 3 h after the irradiation of recipient mice. A group composed of (n = 5) lethally irradiated mice but non-transplanted was included to validate the irradiation source. A control syngeneic group (n = 5) of *Balb/c* recipient mice grafted with *Balb/c* spleen and bone marrow cells was added. GvHD mice were divided into four groups. One group (cGvHD; n = 5 mice) did not receive any macrophage injection. The three remaining groups (n = 10 mice) received a weekly infusion of 2 × 10^6^ intra-peritoneal *Balb/c* PBS-, LPS^low^-, or BCG-trained BMDM, prepared as described above. A total of six injections was performed on D1, D7, D14, D21, D28, and D35 of the experiment. During the early post-irradiation and graft period (day <10), twice mice from the cGvHD-PBS, one mouse from the cGvHD-BCG and two mice from the cGvHD-LPS^low^ group either died spontaneously or were humanly sacrificed because of evident clinical suffering. The remaining animals were sacrificed by cervical dislocation at day 40 after the graft for further analysis.

### Induction of aGvHD in *Balb/c* Mice and Trained Macrophage Infusion

Acute GvHD (aGvHD) was induced in female *Balb/c* mice (H-2d; Janvier Laboratory) by grafting cells from 9- to 10-week-old female fully mismatched major histocompatibility complex (MHC) *C57BL/6* mice (H-2b; Janvier Laboratory), as previously described (30). *Balb/c* mice were lethally irradiated with 750 cGy from a Gammacel [137Cs] source and grafted 3 h later with 5 × 10^6^ RBC-free spleen cells and 1 × 10^6^ BM cells in sterile PBS. Control groups were as follows: a group (n = 5 mice) composed of lethally irradiated mice but non-transplanted and a control syngeneic group (n = 5 mice) of *Balb/c* recipient mice grafted with *Balb/c* spleen and BM cells. Acute GvHD mice were divided in four groups (n = 8 mice each). One group (aGvHD mice) did not receive any macrophage injection. The three remaining groups received a weekly intraperitoneal infusion of 2 × 10^6^ *Balb/c* PBS-, LPS^low^- or BCG-trained bone marrow derived macrophages as previously described. A total of four injections was performed on D1, D7, D14, and D21 of the experiment. Animals were monitored daily for survival and three times a week for weight measurement.

### Graft-*Versus*-Host Disease Monitoring

Monitoring of graft recipients was based on daily observation and measurement of clinical evidence of GvHD (weight loss, manifestations of skin erythema, alopecia, hunching, and diarrhea) and survival during the observation period for both cGvHD and aGvHD experiments. Animals were humanely sacrificed if they had lost more than 40% of their original weight or if they showed evident signs of morbidity and suffering.

### Detection of Anti-DNA Topoisomerase I Autoantibodies in Sera

Levels of anti-DNA topoisomerase I IgG antibodies were assayed by the Scl-70 IgG ELISA kit. Diluted (1/4) mouse serum was distributed into the wells of a microtiter plate (Abnova, Taipei City, Taiwan) coated with purified calf thymus DNA topoisomerase I. The conjugated anti-mice Ig horseradish peroxidase (Dako, Glostrup, Denmark) secondary antibody (1/100) was then added, and the absorbance was read at 450 nm. Results were expressed as antibody index using the cutoff value derived from the Calibrator optical density as previously described (29).

### Serum Alanine Transferase Level

Serum level of ALT was used as a marker of hepatocyte cytolysis and was quantified using a standard clinical automatic analyzer (Modular PP, Roche Diagnostics, Meylan, France).

### Subcutaneous Injection of A20 Cells in *Balb/c* Mice and Trained Macrophage Infusion

The A20 cell line, a *Balb/c* AnN mouse (H-2d haplotype, female genotype) B lymphoma cell line derived from a spontaneous reticulum cell neoplasm (31), was purchased from the American Type Culture Collection (ATTC TIB-208; 1999). A20 cells were expanded in 20 ml culture Flask (Ref 353110, Falcon) in RPMI 1640 culture medium supplemented with 10% FCS, 5 µM *β*-mercapto-ethanol (Ref 31350-010, Gibco) and 1% streptomycin–penicillin.

For tumor challenge, A20 cells were washed twice in RPMI medium and resuspended at a concentration of 2 × 10^6^ cells in 200 µl of PBS and injected in the shaved back of mice. Mice were divided into four groups of n = 7 mice per group. One group (A20-CTRL) did not receive any macrophage injection. The three remaining groups (A20-PBS, A20-LPS^low^, and A20-BCG, respectively) received a weekly intraperitoneal infusion of 2 × 10^6^ *Balb/c* AnN PBS-, LPS^low^- or BCG-trained BMDM as described above. A total of five injections was performed on D1, D7, D14, D21, and D28 of the experiment. Animals were monitored daily for survival and three times a week for weight and tumor measurement. Tumors were measured three times a week with a microcaliper in three dimensions by a scientist blinded to the different treatments, and tumor volumes were calculated as length × width × depth × 0.5236, as previously described (32). Mice that extremely suffered from the tumors and expected to die within the same day of observation (signs including lack of eating/drinking/moving activity under mild stimulation, shivering, eye lids shut, *etc.*) were humanely euthanized on the days of observation. Mice were sacrificed at day 34 of the experiment for further analysis.

### Reverse Transcription-Quantitative PCR

Total mRNA was extracted from crushed samples with TRIzol reagent (Ref 15596018, Ambion). QuantiTect SYBR^®^ Green RT-PCR Kit (Ref 04053228014782, Qiagen) on a LightCycler 480 II instrument (Roche Applied Science, France) was used to perform one-step RT-qPCR. Samples were normalized to mRNA expression of housekeeping genes (GAPDH), and results were expressed as fold increase using the formula 2^−˄˄Ct^. Primers used for PCR are listed in **Supplementary Table S1**.

### Flow Cytometric Analysis of Splenic and Skin-Derived Immune Cells

Cell suspensions (spleen cells, skin-derived immune cells and cell suspensions of the MLR) were incubated for 20 min with 10 µg/ml anti-CD16/CD32 antibody (clone 93, eBiosciences) for Fc receptor saturation and then stained with the appropriate labeled antibody at 4°C for 30 min in the dark in PBS with 2% FBS. The FACS Fortessa II flow cytometer (BD Biosciences) was used to perform flow cytometry according to standard techniques. For spleen and skin characterization of immune cells, the monoclonal antibodies used were B220-Pacific Blue (RA3-6B2, catalog#103230, dilution 1/200), CD4-BV711 (RM4-5, catalog#563726, dilution 1/100), CD8-PE-Cy7 (QA17A07, catalog#155017, dilution 1/200), CD62L-PE-Cy5 (MEL-14, catalog# 104410, dilution 1/200), CD44-APC (IM7, catalog#103012, dilution 1/200), CD44-PeCy7 (IM7, catalog#103029, dilution 1/200), CD69-PercPCy5.5 (H1.2F3, catalog#104522, dilution 1/100), CD11b-BV510 (M1/70C, catalog#101245, dilution 1/200), F4/80-BV711 (BM8, catalog#123147, dilution 1/200), CCR4-BV421 (2G12, catalog#131217, dilution 1/100), CCR5-PercP-Cy5.5 (HM-CCR5, catalog#107015, dilution 1/200), (iCOS ligand) CD278-PeCy5 (15F9, catalog#107708, dilution 1/200), CXCR3-PE Dazzle 594 (CXCR3-173, catalog#126533, dilution 1/100), from BioLegend (Ozyme France, 78180 Montigny-le-Bretonneux), and CCR10-Alexa Fluor 700 (conjugated, catalog#FAB2815N, dilution 1/100) from R&D Systems (RD-BIOTECH, Besancon, France). Data were analyzed with FlowJo software (Tree Star, Ashland, OR).

### ELISA Cytokine Detection

Measurements of IL-2, IL-6, IL-10, IL-13, IL-17A, TNF-*α*,and IFN-*γ* were performed by specific mouse ELISA kits from eBiosciences (Thermo Fisher Scientific, Villebon-Sur-Yvette, France). Concentrations were calculated from a standard curve according to the protocol of the manufacturer.

### Western Blotting of Cell Lysates

Cells were lysed in ice-cold RIPA buffer (10 mmol/L Tris-HCl, pH 7.5; NaCl, 150 mmol/L; 1% Triton X-100; 0.1% sodium dodecyl sulfate) supplemented with 25 mmol/L sodium fluoride, anti-protease 1%, and 0.5 mmol/L sodium orthovanadate. Equal amounts of protein (30 μg) were loaded and separated by 10% sodium dodecyl sulfate-polyacrylamide gel electrophoresis (Biorad Marnes-la-Coquette, France). After transfer and blocking with 5% of fat-free milk and 0.1% Tween in PBS, nitrocellulose membrane was incubated overnight at 4°C with the appropriate dilution of each antibody used according to the guidelines of the manufacturer. The antibodies used in the Western blotting analysis are listed in **Supplementary Table S2**. Specific unconjugated proteins were detected using a 1:10,000 dilution of a horseradish peroxidase-conjugated goat anti-rabbit IgG (Life Technologies, Catalog# 31490) and visualized by an enhanced chemiluminescence system (Advansta, Diagomics, France).

### Histopathological Analysis

Liver, lung, and skin pieces fixed in formol were embedded in paraffin. Tissue sections of 6 mm thickness were made and stained with Sirius red or H&E (hematoxylin and eosin). The slides were examined by standard bright field microscopy (Nikon Eclispe 80i) by a pathologist who was blinded to the experimental group assignment.

### Statistical Analysis

The results were analyzed with the GraphPad Prism5 program. The one-way test ANOVA (Kruskal–Wallis test) was used to verify that the groups were comparable to each other. The Student’s t-test (Mann–Whitney test) was used to determine the differences between two experimental groups. A difference <0.05 was considered as significant.

## Results

### LPS^low^-Trained Macrophages Inhibit T Cell Proliferation in a Mixed Lymphocyte Reaction

**Figure 1:**
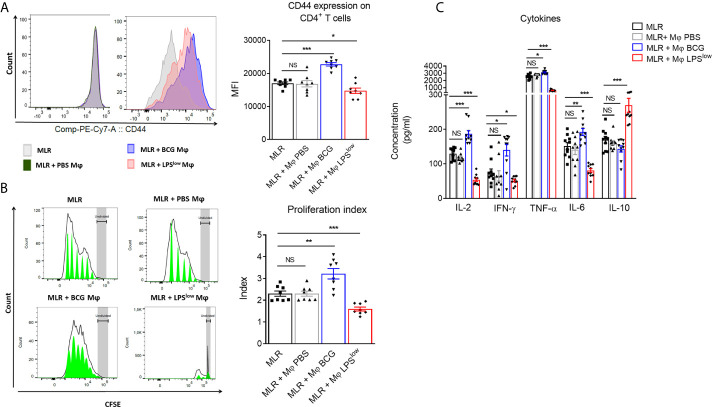
Effects of trained-macrophages adding on CD4^+^ T cell proliferation, activation, and cytokine secretion in a mixed lymphocyte reaction. **(A)** Flow cytometry analysis of CD4^+^ T-cell activation after *in vivo* training during mixed lymphocyte reaction. Responder cells were constituted by purified CD4^+^ T cells derived from *B10.D2* mice. Irradiated splenic cells from *Balb/c* mice constituted the stimulator cells. Cells were cultured in different conditions. Control groups were: CD4^+^ T cells alone in medium, irradiated *Balb/c* splenic cells alone in medium, CD4^+^ T cells with anti-CD3/CD28 antibody. Tested groups were: CD4^+^ T cells with irradiated *Balb/c* (MLR), MLR with PBS-trained *Balb/c* M*φ* (bone marrow derived macrophages), MLR with LPS^low^-trained M*φ*, MLR with BCG-trained M*φ*. Each condition of co-culture was performed in a 10-plicate, and plates were incubated for 4 days at 37°C in 5% CO_2_. Overlaid histograms indicate a representative CD44 expression on gated CD4^+^ T cells of each condition. The column graph represents the mean fluorescence intensity of CD44 expression ± SEM obtained from the 10-plicate of three independent experiments. **(B)** Flow cytometry assessment of CFSE-stained *B10.D2* CD4^+^ T-cell proliferation cultured for 96 h in different conditions. CFSE graphs are representative of each co-culture condition. The first peak represents the undivided cells. The black line represents the different cell populations that underwent division, and the green area shows the peaks of different generations of proliferating cells. The column graph represents the mean of the proliferative index of each condition ± SEM obtained from the 10-plicate of three independent experiments. **(C)** Levels of IL-2, IFN-*γ*, TNF-α, IL-6, and IL-10 cytokines in the supernatant of the different MLR-trained M*φ* conditions. Concentrations were assessed by ELISA and bar graphs represent the mean (pg/ml) ± SEM of 10-plicate of three independent experiments. The ANOVA test with Bonferroni correction was used to detect significant differences between the groups. Statistics are shown between the MLR group and the MLR + trained macrophages conditions. NS, non-significant; *p ≤ 0.05; **p ≤ 0.01; ***p ≤ 0.001.

Trained macrophages can exhibit opposite phenotype and function according to the nature of the first antigenic stimulation (23). Here, we performed a mixed lymphocyte reaction mimicking *in vitro* the allo-immune response involved in the *in vivo* GvHD to evaluate the impact of trained macrophages on the proliferation and cytokine secretion of allogenic CD4^+^ T cells from *B10.D2* mice. The proliferative response was detected by CFSE dilution, and activation of the responder cells was assessed by CD44 surface expression. The CD4^+^ T responder cells showed a strong proliferative response when cultured with irradiated splenic cells from *Balb/c* mice compared to CD4^+^ T responder cells cultured alone. As a positive and a negative control respectively, CD4^+^ T cells stimulated with anti-CD3/CD28 antibodies showed a strong proliferation index and a high activation status while irradiated *Balb/c* splenic cells barely proliferated (**Supplementary Figures S1A, B** **)**. The addition of PBS-macrophages to the allogeneic reaction did not influence the proliferative response and activation of the allogeneic CD4^+^ T responder cells. By contrast, CD4^+^ T responder cells showed an enhanced proliferative index (p = 0.01), associated with increased CD44 expression (p = 0.0002) when cultured with BCG-macrophages, while their proliferation as well as activation was significantly reduced when cultured with LPS^low^-macrophages (p = 0.0009 and p = 0.003, respectively, **Figures 1A, B** **)**. We then assessed the impact of trained macrophages on the function of allogeneic CD4^+^ T responder cells by measuring the levels of IL-2, IFN-*γ*, TNF-*α*, IL-6, and IL-10 in the supernatant of the different MLR conditions. Basal cytokine production of the irradiated *Balb/c* splenocytes and the *B10.D2* purified CD4^+^ T cells (either unstimulated or stimulated with anti-CD3/CD28 antibodies) is shown in **Supplementary Figure S1C**. Of note, analysis of cytokine production in the supernatant of MLR with PBS-trained macrophages condition did not show any difference compared to the MLR condition alone. Addition of BCG-macrophages was associated with a significant increase in IL-2 (p < 0.0001), IL-6 (p = 0.009), TNF-α (p = 0.01), and IFN-*γ* (p = 0.01) in the supernatant, compared to the allogeneic MLR condition (**Figure 1C**). On the contrary, the addition of LPS^low^-macrophages in the MLR significantly reduced the levels of IL-2 (p < 0.0001), IL-6 (p < 0.0001), TNF-α (p = 0.0008), and IFN-*γ* (p = 0.02) while significantly increasing IL-10 in the culture supernatants (p = 0.0004, **Figure 1C**). Collectively, these data suggest that appropriately trained macrophages can act as either an enhancer or a suppressor of allogeneic CD4^+^ T cell proliferation, activation, and cytokine response in the setting of MLR.

### Adoptive Transfer of Trained Macrophages Modulates cGvHD in Mice

**Figure 2:**
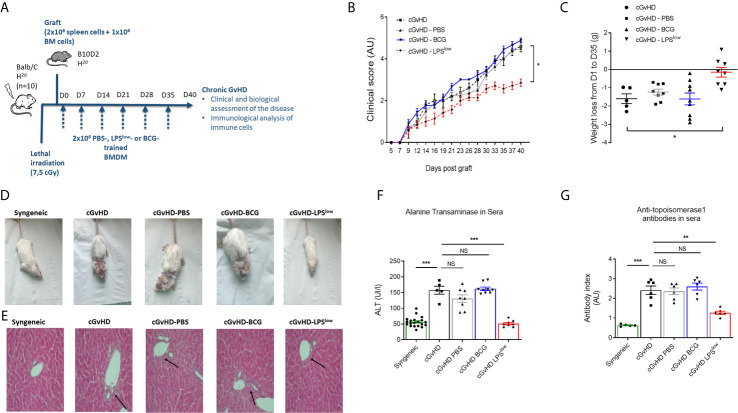
Adoptive transfer of trained macrophages modulates cGvHD in mice. **(A)** Schematic representation of the experimental induction of sclerodermatous chronic graft-*versus*-host disease in mice and scheduling of trained macrophages infusion. Mice (n = 7 at least per group) were monitored for survival, weight, clinical signs of chronic GvHD for 40 days in one experiment. **(B)** Clinical score assessment of the different groups of cGvHD mice. The scoring system included five clinical criteria (maximum index of 5) representing the sum of the recorded values and expressed in arbitrary unit (AU). **(C)** Weight loss assessment of the different groups of cGvHD mice. Mice were weighted at day 0 of the experiment and equitably distributed into the different groups according to their initial weight. At day of the sacrifice, each identified mouse was weighted to determine the weight loss delta. Scatter plots represent individual data with mean ± SEM obtained from at least n = 5 biologically independent mice: syngeneic (n = 5); cGvHD (n =5); cGvHD-PBS (n = 8); cGvHD-BCG (n = 9); cGvHD-LPS^low^ (n = 8). Statistical differences between the groups were detected by the ANOVA test with Bonferroni correction. **(D)** Representative photographs of mice at day 40 after BMT showing hair aspect (spiky coat and alopecia in the cGvHD animals compared to the syngeneic group). **(E)** Representative liver section of 5 μm stained with hematoxylin and eosin showing increased periportal mononuclear cell infiltration in cGhVD mice compared to syngeneic group. Photographs were taken with a Nikon Eclipse 80i microscope. Original magnification ×40. **(F)** Levels of serum alanine aminotransferase (ALT) in the different experimental groups. Each box represents the mean of the concentration expressed in international unit per liter ± SEM. **(G)** Anti-topoisomerase 1 antibody levels in the different experimental groups measured by ELISA. Each box represents the mean of the antibody index expressed in arbitrary unit ± SEM. The ANOVA test with Bonferroni correction was used to detect significant differences between the groups (unless stated). NS, non-significant; *p ≤ 0.05; **p ≤ 0.01; ***p ≤ 0.001.

We next investigated the *in vivo* effects of the adoptive transfer of trained macrophages on the course of cGvHD. We used the well-established animal model of sclerodermatous cGvHD triggered upon transplantation of *B10.D2* (H-2d) BM and spleen cells across minor histocompatibility loci into lethally irradiated *Balb/c* (H-2d) recipients (**Figure 2A**). Clinical score assessment started 10 days after transplantation. At day 40, cGvHD mice had a score of 4.60 ± 0.36 while syngeneic remained null (p < 0.0001). Weight loss was marked in cGvHD mice compared to syngeneic animals (p = 0.005, **Figure 2B**). Importantly, infusion of LPS^low^-macrophages significantly attenuated the severity of the clinical score and the weight loss compared to the untreated mice (p = 0.005 and p = 0.006, respectively, **Figures 2B, C** **)**. Adoptive transfer of PBS-macrophages did not affect the clinical score nor the weight loss, whereas BCG-macrophage infusion tended to aggravate them though not significantly (p = 0.15 and p = 0.23, respectively). Representative photographs of mice at day 40 after BMT showed spiky coat and alopecia in the cGvHD animals with a favorable resolution in the cGvHD-LPS^low^ group (**Figure 2D**). Histological hematoxylin–eosin staining showed mixed lymphomonocytic infiltrations around the portal vein, which was significantly suppressed in the cGvHD-LPS^low^ group (**Figure 2E**). While mice receiving PBS or BCG-macrophages did not significantly affect cGVHD-induced increase in serum ALT, a marker of GvHD-associated liver injury, a virtually complete inhibition was observed in cGvHD-LPS^low^ group compared to the cGVHD group (p < 0.0001) (**Figure 2F**).

**Figure 3:**
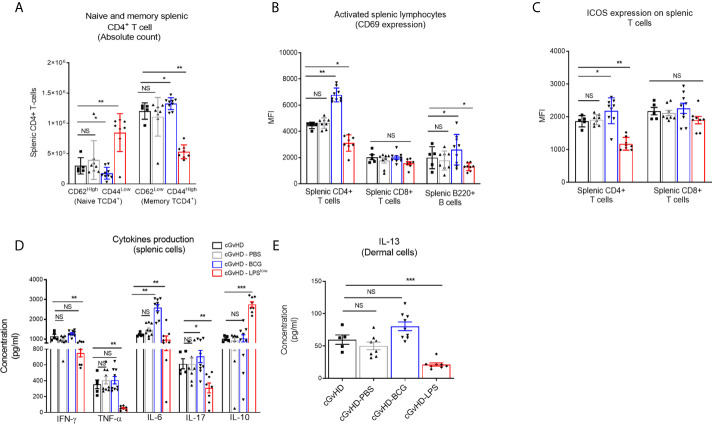
Trained macrophage infusion affects the activation and the cytokine production of splenic immune cells in cGvHD mice. **(A)** Frequency of naive and memory CD4^+^ T-cell subpopulations in the spleen of the different experimental groups. Data represent absolute count of naive (CD62L^High^ CD44^Low^) and memory (CD62L^Low^ and CD44^High^) CD4^+^ T cells among the total splenic CD4^+^ T-cell population ± SEM obtained from at least n = 7 biologically independent mice. **(B, C)** Activation of splenic CD4^+^, CD8^+^ T cells and B220^+^ B cells assessed by flow cytometric measurement of the CD69 expression **(B)** and ICOS expression for the T-cell subpopulations **(C)**. Data represent the MFI ± SEM from at least n = 7 biologically independent mice. **(D)** Cytokine production in the supernatant of stimulated splenic cells. Spleen cell suspensions were stimulated with 5 μg ml^−1^ of Concanavalin A and incubated for 48 h at 37 °C with 5% CO_2_. Levels of IFN-*γ*, TNF-*α*, IL-6, IL-17, and IL-10 were assessed by ELISA, and concentrations are expressed in pg/ml. Data represent the mean ± SEM from the duplicate of at least n = 7 biologically independent samples. **(E)** ELISA assessment of IL-13 production (pg/ml) by skin-derived immune cells. Each box represents mean ± SEM of triplicate obtained with cell culture from at least n = 7 biologically independent mice. NS, Non-significant; *p ≤ 0.05; **p ≤ 0.01; ***p ≤ 0.001.

Chronic GvHD is characterized by dysregulated adaptive immune response with an inappropriate activation of allogeneic CD4^+^ T cells that leads to an alteration of B and CD8^+^ T-cell responses, responsible for the autoimmune features and tissue damage (33, 34). Anti-DNA topoisomerase I autoantibody production, indicative of this immune dysregulation, was increased in cGvHD mice compared to the syngeneic mice (p < 0.001, **Figure 2G**). Importantly, LPS^low^-macrophages induced a significant reduction in serum anti-topoisomerase 1 antibody index compared to the untreated cGvHD mice (p = 0.002), while the adoptive transfer of PBS- and BCG-macrophages had no affect (**Figure 2G**). We then assessed the immunological effects of the infused macrophages on the course of the cGvHD. Activation of T and B cells as well as a shift toward a predominant memory phenotype of CD4^+^ T cells is a hallmark of established cGvHD (35) (**Supplementary Figures S2A–C**). As shown in **Figures 3A–C**, adoptive transfer of LPS^low^-macrophages significantly reduced CD69 expression on CD4^+^ T and B cells (p = 0.02 and p = 0.005) but not on CD8^+^ T cells and was associated with a reduced frequency of memory CD4^+^ CD62L^Low^ CD44^High^ T cells compared to untreated cGvHD mice (p = 0.006). By contrast, BCG-macrophage treatment increased the activation of CD4^+^ T cells (p = 0.004) and significantly augmented the proportion of memory over naïve CD4^+^ T cell subpopulation (p = 0.01). The inducible costimulatory molecule ICOS (CD278), which participates in the augmented T-cell response during cGvHD (36) was downregulated in cGvHD-LPS^low^ mice (p = 0.001) but enhanced in cGvHD-BCG group (p = 0.04, **Figure 3C**).

### cGvHD-Associated Dysregulation of Cytokine and Chemokine Production Is Alleviated Upon Adoptive Transfer of LPS^low^-Macrophages

Cytokine network in the cGvHD is adversely altered, with an early increase in Th1 and Th17 pro-inflammatory cytokine production, but a predominance of the Th2 subset at the chronic stage (37) (**Supplementary Figures S2D, E** **)**. Spleen cells from cGvHD-LPS^low^ had a significant reduction in TNF-*α* (p = 0.003), IL-6 (p = 0.006), IFN-*γ* (p = 0.009), and IL-17 (p = 0.007) production but a concomitant increase in IL-10 secretion (p = 0.0005, **Figure 3D**). In contrast, splenic cells from cGvHD mice treated with BCG-macrophages (cGvHD-BCG) displayed a heightened capacity to produce the pro-inflammatory cytokine IL-6 (p = 0.005) and IL-17 (p = 0.03) compared to the untreated cGvHD mice (**Figure 3D**). Furthermore, dermal cells from cGvHD-LPS^low^ animals showed a significantly reduced capacity to secrete IL-13 compared to the cGvHD mice (p = 0.0008), which was not affected by infusion with BCG-macrophages (**Figure 3E**).

**Figure 4:**
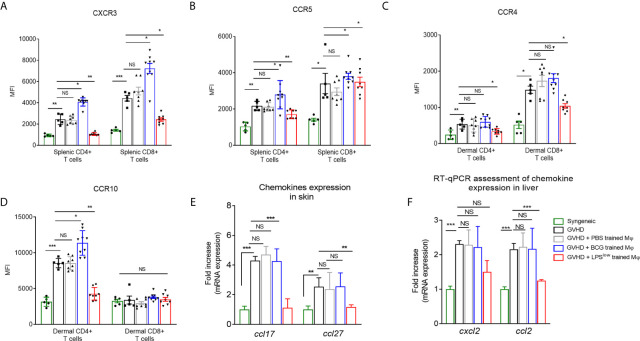
LPS^low^-macrophage injections influence the chemokine network in cGvHD mice. **(A, B)** Flow cytometric analysis of splenic CD4^+^ and CD8^+^ T-cells CXCR3 and CCR5 expression at day 40. **(C, D)** Flow cytometric analysis of dermal CD4^+^ and CD8^+^ T-cells CCR4 and CCR10 expression at day 40. Gating strategy is represented in **Supplementary Figure S4**. Data represent the mean fluorescence intensity of the chemokine receptors expression on T cells obtained from at least n = 7 biologically independent mice. The ANOVA test with Bonferroni correction was used to detect significant differences between the groups. NS, non-significant; *p ≤ 0.05; **p ≤ 0.01; ***p ≤ 0.001. **(E, F)** RT-qPCR assessment of c*cl17* and c*cl27* mRNA levels in the skin **(E)** and *cxcl2* and *ccl2* mRNA levels in the liver **(F)** of cGvHD mice at day 40 of the experiment. Results were expressed as fold increase *versus* the syngeneic group, derived from at least n = 7 biologically independent mice. Unpaired T-test was used to detect the significance between the groups. NS, non-significant; *p ≤ 0.05; **p ≤ 0.01; ***p ≤ 0.001.

Next, we set out to decipher whether the trained-macrophage infusion could affect the expression of chemokine receptors on allogeneic T cells that play an important role in lymphocyte migration to the sites of allo-recognition and priming (38). Chronic GvHD development was accompanied by an upregulation of CCR5 and CXCR3 on splenic T CD4^+^ and CD8^+^ cells and increased frequency and expression of CCR10 and CCR4 on dermal CD4^+^ T cells (**Figures 4A–D**). Of note, infusion of LPS^low^-macrophages significantly downregulated the expression of CXCR3 (p = 0.001 and p = 0.03) and CCR5 (p = 0.005 and p = 0.03) on splenic CD4^+^ and CD8^+^ T cells; the expression of these chemokine receptors was strongly induced by adoptive transfer of BCG-macrophages (**Figures 4A, B** **)**. Similarly, at the dermal level (**Figures 4C, D** **)**, LPS^low^-macrophage transfer significantly diminished CCR4 expression on CD4^+^ and CD8^+^ T cells (p = 0.02 and p = 0.03), whereas CCR10 was only decreased on CD4^+^ T cells (p = 0.001). cGvHD-BCG mice had an increased expression of only CCR10 on their dermal CD4^+^ T cells (p = 0.03). In parallel, we evaluated the dermal mRNA expression of the skin homing chemokines *ccl17* and *ccl27* and observed that they were increased in cGvHD group compared to the syngeneic mice (p < 0.0001). As shown in **Figure 4E**, BCG-macrophage infusion only upregulated the expression of *ccl27* (p = 0.04), while LPS^low^-macrophages abolished both the overexpression of *ccl17* (p = 0.005) and *ccl27* (p = 0.003). Finally, we analyzed the mRNA expression of two chemokines that promote the migration of the leukocyte subsets to the liver, *ccl2*, and *cxcl2* (39, 40). Untreated cGVHD mice exhibited an increase of hepatic *ccl2* and *cxcl2* compared to syngeneic grafted mice (p < 0.0001). A downregulation of *ccl2* (p = 0.004) and trend towards a decrease of *cxcl2* expression (p = 0.18) were observed in the liver of cGvHD-LPS^low^ mice. Neither PBS- nor BCG-trained macrophage injections have a significant impact on the *ccl2* and *cxcl2* expression in the hepatic tissue (**Figure 4F**).

Altogether, these results indicate that infusion of LPS^low^-macrophages exerts a suppressive effect on allogeneic responses during cGvHD with a drop in inflammatory cytokines and chemokine expression in target organs, but increased IL-10 production and a downregulation of chemokine and activation receptors on allogeneic T cells.

### LPS^low^-Macrophages Serve as Protective Barrier Against cGvHD-Induced Tissue/Organ Injury

**Figure 5:**
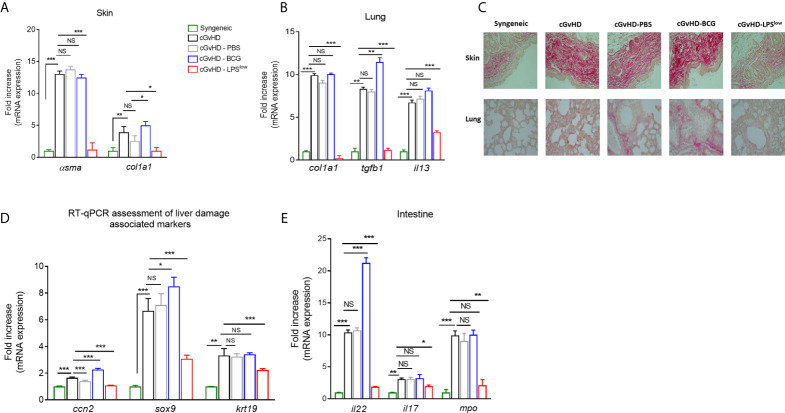
LPS^low^-macrophages serve as protective barrier against cGvHD-induced tissue/organ injury. **(A, B)** RT-qPCR assessment of *α-sma* and c*ol1a1* mRNA levels in the skin **(A)** and of c*ol1a1*, *tgfb1* and *il13* mRNA levels in the lungs **(B)** of cGvHD mice at sacrifice. Results were expressed as fold increase *versus* the syngeneic group, derived from at least n = 7 biologically independent mice. **(C)** Representative Sirius red-dyed skin and lung sections of 5 μm, showing enhanced fibrosis with accumulation of collagen in the dermis and thickening of inter-alveolar septa reflecting the obliterative bronchiolitis of cGvHD mice compared to syngeneic mice. Photographs were taken with a Nikon Eclipse 80i microscope. Original magnification ×40. **(D, E)** RT-qPCR assessment of *il22*, *il17*, and *mpo* mRNA levels in the intestines **(D)** and of *ccn2*, *sox9*, and *krt19* in the liver tissue **(E)** of cGvHD mice at day 40 of the experiment. Results were expressed as fold increase *versus* the syngeneic group, derived from at least n = 7 biologically independent mice. Unpaired T-test was used to detect the significance. NS, non-significant; *p ≤ 0.05; **p ≤ 0.01; ***p ≤ 0.001.

Chronic GvHD is accompanied by serious skin and visceral injuries including fibrotic lesions of the lung and severe intestinal crypt damage (41). As a next step to evaluate the *in vivo* effects of infusion of trained macrophages on the visceral involvement of cGvHD, we quantified the mRNA expression of the fibrosis-associated type I collagen *Col1a1* and *α*-smooth muscle actin genes in skin and lungs. As expected, we observed an upregulation of mRNA expression of *Col1a1* and *α-sma* in skin and lungs in the cGvHD group compared to the syngeneic mice (p < 0.0001). BCG-macrophage infusion increased the expression of *Col1a1* in skin (p = 0.03) while cGvHD-LPS^low^ mice showed a significant reduction of *Col1a1* (p = 0.02 and p = 0.0008) and *α-sma* mRNA expression in skin and lungs (p = 0.0007 and p = 0.0006, respectively, **Figures 5A, B** **)**. Moreover, cGvHD was accompanied by an upregulation of *il13* mRNA expression in lungs compared to the syngeneic group (p < 0.0001). LPS^low^-macrophages attenuated *il13* expression (p = 0.0009) while the BCG-macrophages did not have any effect compared to the cGVHD group (**Figure 5B**). Sirius red-dyed staining of dermal and pulmonary sections showed clear evidence of collagen accumulation driven by the cGvHD. Infusion with LPS^low^-macrophages resulted in alleviated fibrosis in skin and lungs (**Figure 5C**). We then analyzed the expression of *ccn2*, a matricellular protein that is upregulated in fibrotic hepatic disorders (42) and *sox9/krt19* expression, markers associated with chronic liver damage (43). We observed an increased expression of *ccn2*, *sox9*, and *krt19* in cGvHD mice compared to syngeneic (p = 0.03, p = 0.002, and p = 0.007 respectively). LPS^low^-macrophage treatment diminished the expression of the assessed markers, while BCG-macrophage injection worsened the expression of *ccn2* and *sox9* compared to the cGVHD mice (**Figure 5D**). Lastly, the intestinal manifestation of cGvHD was assessed by mRNA quantification of *il22* and *il17* for the barrier integrity and *mpo* expression for quantification of the inflammatory infiltrate. As expected, cGvHD mice showed a significant upregulation of *il22* (p = 0.0008), *il17* (p = 0.007), and *mpo* (p = 0.0007) genes compared to the syngeneic group. Results showed that infusion with BCG-macrophages exacerbated the expression of *il22* (p = 0.0008), whereas the adoptive transfer of LPS^low^-macrophages significantly reduced mRNA levels of *il22*, *il17*, and *mpo* (p = 0.0007, p = 0.03, and p = 0.009, respectively) (**Figure 5E**).

Collectively, these findings show the beneficial effects of LPS^low^-macrophages on the onset and clinical features of cGvHD and highlight the correlation between the specific immunological modifications and the impact on the visceral manifestation of the disease.

### Weekly Infusion of LPS^low^-Trained Macrophages Alleviates Acute GvHD in Mice

**Figure 6:**
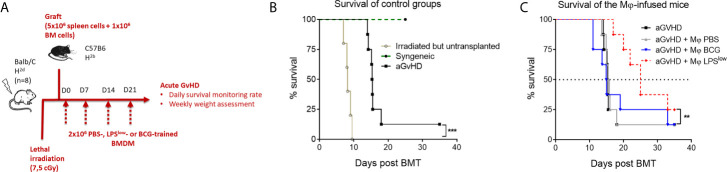
Weekly infusion of LPS^low^-trained macrophages alleviates acute murine GvHD. **(A)** Schematic representation of the *in vivo* induction of MHC-mismatched acute GvHD and scheduling of trained macrophages infusion. Mice (n = 8 per group) from one experiment were monitored for survival. **(B)** Kaplan–Meier survival curve of the control groups of the acute GvHD experiment showing the survival rate of syngeneic, irradiated, but untransplanted and aGvHD mice. **(C)** Survival rate of the different groups of the experiment. Each group: aGvHD, aGvHD + M*φ* PBS, aGvHD + M*φ* BCG, aGvHD + M*φ* LPS^low^ contained (n = 8) mice. Statistics are shown between the non-treated aGvHD and the aGvHD + M*φ* LPS^low^ group. The log-rank (Mantel–Cox) and the Gehan–Breslow–Wilcoxon statistic tests were used to detect the differences between the groups. NS, non-significant; **p ≤ 0.01; ***p ≤ 0.001.

Acute GvHD was induced using the fully mismatched MHC mouse model of hematopoietic stem cell transplantation of lethally irradiated *Balb/c* recipient mice with splenocytes and bone marrow cells from *C57BL/6* mice (**Figure 6A**). Survival rate was monitored daily. As shown in **Figure 6B**, lethally irradiated but non-transplanted mice had a median survival of 8 days, while all mice from the syngeneic group remained alive at the end of the experiment (day 30). Mice started to lose weight after the irradiation, but all transplanted animals regained weight after bone marrow transplantation (BMT). Allogeneic-transplanted mice started to lose weight around day 10 and reached the nadir at day 25. Clinical signs of acute GvHD started at day 11 of the experiment with decreased activity, hunched posture, and progressive alopecia (**Figure 6C**). Daily monitoring showed that aGvHD mice infused with LPS^low^-macrophages had a trend towards an improved survival rate compared to the untreated group. PBS and BCG-macrophage treatment did not influence the survival rate of the aGvHD mice (**Figure 6C**). Thus, adoptive transfer of LPS^low^-macrophages may exert a protective effect against acute GvHD, similar to what was observed with the cGvHD model.

### LPS^low^-Trained Macrophages Hamper Allogeneic T-Cell Activity in an IL-10-Dependent Manner

**Figure 7:**
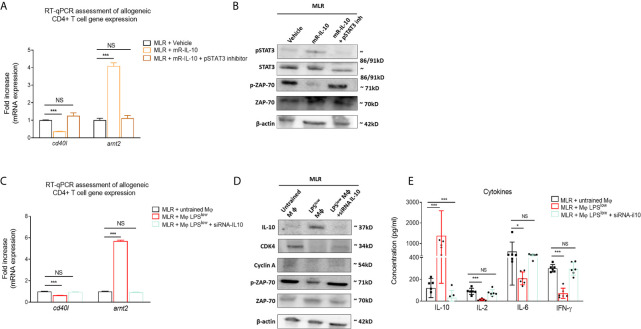
LPS^low^-trained macrophages hamper allogeneic T-cell activity in an IL-10-dependent manner. **(A, B)** Purified CD4^+^ T cells derived from B10.D2 mice co-cultured with irradiated splenic cells from *Balb/c* mice were treated with 10 µM of STAT3 inhibitor V or vehicle (PBS + 1%DMSO) for about 30 min before mouse recombinant IL-10 stimulation for 24 h. **(A)** RT-qPCR assessment of *arnt2* and *cd40l* mRNA expression. **(B)** Western blot analysis of pSTAT3, STAT3, pZAP70, ZAP-70, and *β*-actin expression. Representative results of two experiments performed. mR-IL-10, mouse recombinant IL-10. **(C, D)** Bone marrow derived macrophages obtained from *Balb/c* mice were either stimulated by PBS (untrained) or trained with LPS^low^, and subjected to IL-10 mRNA invalidation with small interfering RNA. Cells were then co-cultured with allogeneic CD4^+^ T cells from the MLR for 72 h in six-plicate. Cells were then analyzed by RT-qPCR for *cd40l* and *arnt2* mRNA expression **(C)** and by Western blot for IL-10, CDK4, Cyclin A, pZAP70, ZAP-70, and *β*-actin expression **(D)**. Representative results of two experiments performed. **(E)** ELISA measurement of IL-10, IL-2, IL-6, and IFN-*γ* levels in the supernatant of the allogeneic CD4^+^ T cells co-cultured with wild-type untrained macrophages, LPS^low^ -macrophages, or IL-10 KO LPS^low^-macrophages. Data represent the mean (pg/ml) ± SEM of six-plicate of two independent experiments. Unpaired T-test and the ANOVA test with Bonferroni correction were used to detect the significance. NS, non-significant; *p ≤ 0.05; ***p ≤ 0.001.

We have shown in the first experiment that LPS^low^-macrophage addition to the MLR system was associated with a marked increase of IL-10 level in the supernatant. Thus, we focused on that cytokine to get further into the mechanistic features of the protective effect of LPS^low^-macrophages in the aGvHD and cGvHD models. We showed that adding recombinant mouse IL-10 (rm-IL-10) cytokine in the MLR system upregulated the phosphorylation of STAT3 and the mRNA expression of the IL-10 target gene *arnt2 *(44) but decreased the phosphorylation of T-cell specific protein kinase ZAP-70 (45) and the mRNA expression of the T-cell activation marker cd*40l* (46) (**Figures 7A, B** **)**. Similarly, co-culture of LPS^low^-macrophages with allogeneic CD4^+^ T cells increased *arnt2* expression but was associated with a decrease of *cd40l* mRNA level (**Figure 7C**), a downregulation of the proliferation and cell-cycle associated proteins CDK4 and Cyclin A (**Figure 7D**), and a drop of IL-2, IL-6, and IFN-*γ* levels in the supernatant of the MLR milieu (**Figure 7E**). Notably, the inhibition of the STAT3 signaling pathway (**Figures 7A, B** **)**, as well as the mRNA IL-10 silencing of the LPS^low^-macrophages co-cultured with the allogeneic CD4^+^ T cells abrogated the effects observed with wild-type LPS^low^-macrophages (**Figures 7C–E**). Altogether, these results highlight the involvement of IL10 in the modulation of the allogeneic response of CD4^+^ T cells and deliver further mechanistic insights into the immunomodulatory effect of LPS^low^-macrophages in the aGvHD and CGvHD models.

### Adoptive Transfer of LPS^low-^Trained Macrophages Does Not Exacerbate B Lymphoma in Mice

**Figure 8:**
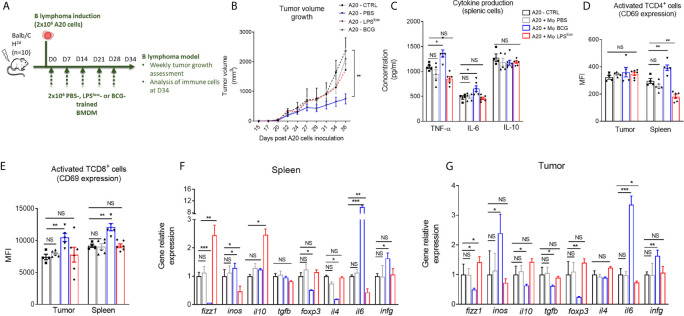
Clinical and immunological impact of the adoptive transfer of trained-macrophages on A20 cells B lymphoma tumor growth in mice. **(A)** Schematic representation of the *in vivo* induction of B lymphoma in mice and scheduling of trained macrophage infusion. Mice (n = 7 in each group) were subcutaneously injected with 2 × 10^6^ A20 cells and sacrificed at day 34 in one experiment. **(B)** Tumor volume evolution in the different groups of A20-challenged mice (n = 7). Tumor growth was monitored three times a week with a microcaliper, and tumor volumes were calculated as length × width^2^ × 0.5236 and expressed in mm^3^. Statistics are shown between the BCG-macrophages infused (A20-BCG) and the untreated A20 groups. Differences were detected at sacrifice using the Mann–Whitney test. **p ≤ 0.01. **(C)** Cytokine production in the supernatant of splenic cells harvested at sacrifice, stimulated with 5 μg/ml of Concanavalin A and incubated for 48 h at 37 °C with 5% CO_2_. Levels of TNF-α, IL-6, and IL-10 were assessed by ELISA and concentrations are expressed in pg/ml. Data represent the mean ± SEM from the duplicate of at least n = 5 biologically independent samples. **(D, E)** Flow cytometric analysis of splenic and tumoral CD4^+^ **(C)** and CD8^+^ **(D)** T-cell phenotype at day 34 of the experiment. Tumor infiltrating T cells were gated on CD3 and CD4 or CD8 positive cells among CD45 positive population. Data represent the mean fluorescence intensity of CD69 ± SEM, reflecting the activation status of the assessed cells, obtained from at least n = 5 biologically independent samples. **(F, G)** RT-qPCR assessment of *fizz1*, *inos*, *il10, tgfb*, *foxp3*, *il4*, *il6*, and *infg* mRNA levels in the spleen and tumor lesions of the different groups of A20-challenged mice at day 36 of the experiment. Results were expressed as fold increase *versus* the untreated A20-mice group, derived from at least n=5 biologically independent samples. Unpaired T-test was used to detect the differences between the groups. NS, non-significant; *p ≤ 0.05; **p ≤ 0.01; ***p ≤ 0.001.

It is well established that therapeutic strategies used for the prophylaxis and treatment of GvHD can diminish the beneficial effect of graft-*versus*-leukemia effects (4). Hence, we evaluated whether the protective/inhibitory effects of LPS^low^-macrophages on the course of acute or chronic GvHD observed in our study could influence tumor progression in a B lymphoma (A20) murine model. Mice were injected subcutaneously with 10^6^ A20 cells and subjected to weekly infusion of trained macrophages (**Figure 8A**). Tumors were detected as early as day 10 of the injections. During the experiment (34 days), only three animals died spontaneously as a result of rapid tumor progression, and for ethical reasons, two were humanly euthanized at day 28 when the tumor reached a pre-defined volume size of 3,000 mm^3^. As shown in **Figure 8B**, tumors expanded rapidly in the different groups, though less importantly in the A20-BCG group. At the end of the experiment (day 34), no difference in tumor volume was detected between the A20-CTRL, the A20-PBS, and the A20-LPS^low^ groups. Interestingly, A20-BCG mice had significantly reduced tumor volume at day 34 compared to the other groups (p = 0.03). As a next step to decipher the effects of the infusion of trained macrophages observed in this experiment, we assessed the activation status and cytokine production by splenic and tumor infiltrating T cells. BCG-macrophage treatment resulted in an increase in TNF-α and IL-6 production by splenic cells compared to untreated A20 mice (p = 0.03 and p = 0.04, respectively), while PBS and LPS^low^-macrophage infusions had no effects. Splenic IL-10 production capacity remained unchanged in the four groups (**Figure 8C**). Activation of CD4^+^ and CD8^+^ T cells was assessed by CD69 expression at both the systemic level on the spleen and *in situ* on tumors. BCG-macrophage treatment enhanced the activation of both CD4^+^ and CD8^+^ *in situ* (p = 0.0006 and p = 0.0008), while it only activated CD4^+^ T cells in the spleen (p = 0.006, **Figure 8D**). The mean fluorescence intensity of CD69 on CD4^+^ and CD8^+^ T cells in spleen and tumors did not differ in untreated A20 mice or in mice infused with PBS or LPS^low^-macrophages (**Figure 8E**). Finally, to get further into the molecular changes underlying the effects of the infused macrophages on the tumor growth, we analyzed the expression of several genes involved in both the anti-tumoral and the allogenic immune responses (**Figures 8F, G** **)**. PBS-macrophage infusion did not induce any change in gene expression in the isolated A20 tumors or in the spleen tissues compared to the untreated A20-CTRL group. A20 mice infused with LPS^low^-macrophages upregulated the expression of *fizz1* (found in inflammatory zone-1) gene which is usually associated with the alternatively activated macrophages phenotype (M2 macrophages), in the spleen (p = 0.0005) and in the tumor (p = 0.03) and *il10* gene only in the spleen (p = 0.02) compared to untreated A20 mice. Moreover, LPS^low^-macrophages downregulated the expression of *inos* in spleen (p = 0.03) and *il6* both in spleen (p = 0.005) and the tumor (p = 0.02). All the other genes assessed remained unchanged. BCG-macrophage infusion was associated with an upregulation of the splenic and tumoral expression of *inos* (p = 0.03), *il6* (p = 0.0005), and *infg* (p = 0.03), markers that are associated with classically activated macrophage phenotype (M1 macrophages). By contrast, we observed a decrease in the expression of *fizz1* (p = 0.01) and the T regulator cell transcription factor *foxp3* (p = 0.03) in both the spleen and the tumor tissues. Furthermore, A20-BCG mice had a diminished *il4* (p = 0.03) expression in spleen and a drop in *il10* and *tgfb* expression in tumors (p = 0.03 and p = 0.02, respectively).

Cumulatively, these results show that LPS^low^-macrophages do not adversely affect tumor growth and are not accompanied by major modifications of T cell activation, cytokine production, or gene expression. Interestingly, infusion with BCG-macrophages exhibit an anti-tumoral activity with an upregulation of the M1-associated genes and an increase of T-cell activation and pro-inflammatory cytokine secretion.

## Discussion

Immunological memory is traditionally associated with the adaptive immune system. Yet, this dogma has been recently challenged as innate immune cells like macrophages can be trained to mount a heightened inflammatory response upon non-specific antigenic challenge, or on the contrary can be rendered hypo-responsive with immunosuppressive effects (11, 47). In this study, we show that LPS^low^-macrophages are efficient in targeting inflammation-mediated allo-immune responses associated with acute and chronic GvHD, resulting in significant disease alleviation. On the contrary, BCG-macrophages tend to aggravate the clinical and biological features of cGvHD. The pathophysiology of GvHD is complex, highly dependent on major histocompatibility complex (MHC) antigenic disparity between donor and recipient, and involves a variety of cell types in both its generation and subsequent pathologic manifestations (48). The release of pro-inflammatory cytokines from activated immune cells including macrophages and donor T allo-reactive cells lies at the center of GvHD pathogenesis (41). This process enhances the interaction between antigen presenting cells and donor-derived lymphocyte populations, including CD4^+^ T cells, resulting in the generation of effector populations (6). As a first step to decipher the immunomodulatory effects of trained macrophages in our *in vivo* GvHD model, we reproduced *in vitro* the onset of the GvHD allo-immune reaction using a minor MHC mismatched MLR. LPS^low^-macrophage addition limited the activation and proliferation of CD4^+^ T cells, along with a decrease of IL-2, IFN-*γ*, TNF-*α* and IL-6 production but an increase of IL-10 release, while BCG-macrophages had an opposite effect. T-cell receptor (TCR) recognition of the MHC-allo antigen complex and costimulatory molecule engagement results in IL-2 release by CD4^+^ T cells that exerts a potent proliferative and growth autocrine effect (49). These activated allogeneic CD4^+^ T cells upregulate CD44 expression that binds to the lymphocyte-specific protein kinase (Lck) and thereby enhances T-cell receptor signaling (50). During the allo-immune response, activated T cells undergo various polarization status influenced by the exogenous cytokine milieu (51), which generates heterogeneous cell populations predominated by the IFN-*γ* Th1 releasing subtype that triggers the initial effector mechanisms (52). Studies on innate immune memory demonstrated that BCG-training of macrophages results in upregulation of pattern recognition receptors and costimulatory molecules with heightened non-specific production of the pro-inflammatory cytokines, IL-6, and TNF-*α* (13, 23). IL-6 has anti-apoptotic properties as it prolongs CD4^+^ T cell survival *in vitro* by inducing Bcl-2 expression in the T cells (53) and downregulating FasL expression (54). Furthermore, TNF-*α* has the capacity to promote TCR-dependent T-cell activation (55) *via* the activation of NF*κ*B pathway (56). Conversely, the immunoregulatory IL-10 cytokine, the expression of which is upregulated in LPS^low^-macrophages following inflammatory stimulation (10, 23, 57), reduces MHC class II expression on monocytes/macrophages, inhibits T-cell proliferation, and abrogates Th1, Th2, and Th17 cytokine production (58). In addition, LPS^low^-trained macrophages have been shown to adopt a hypo-responsive phenotype with a downregulation of the co-stimulatory molecules such as CD80, inflammatory markers (Ly6C) but an upregulation of the mannose receptor CD206 and the DC-SIGN (CD209). These phenotypes persist *in vivo* after peritoneal injections and can be also transferred to splenic and peritoneal macrophages (23, 59).

Our findings indicate that macrophages acquire a modified phenotype according to the nature of immune training and thus react differently once in contact with the allogeneic T cells, either to promote or to inhibit the allo-immune response.

Chronic inflammation, fibrosis, and autoimmune disorders are the pathologic hallmark of cGvHD (60). We therefore used the murine sclerodermatous cGvHD model that mimics clinical and biological features of the human chronic form, involving both inflammatory and pro-fibrotic components (5). In the first stage of the disease, tissue damage through irradiation results in the upregulation in TNF-*α*, IL-1β, and IL-6 inflammatory cytokines that favors activation of host dendritic cells and monocytes/macrophages. In addition, recognition of the host minor antigens by donor CD4^+^ T cells leads to an activation of a Th1 response that drives the initial inflammatory reaction with an early activation of the Th17 lymphocyte subset, known to exert potent chemoattractant effects and promote the recruitment of T lymphocytes in target tissues (61). This initial inflammatory reaction is followed by a progressive shift toward the Th2 response with IL-13 and IL-4 production that drives the activation of profibrotic macrophages through TGF-*β* signaling (62, 63) and induces hyperactivation of the autoreactive B cells responsible for the autoimmune features of the disease (64). The major finding of this study was that LPS^low^-macrophage infusion attenuated the development of sclerodermatous cGvHD, while BCG-macrophages tended to aggravate it. Consistent with our *in vitro* results, we observed a drop in splenic TNF-*α*, IL-6, IFN-*γ*, and IL-17, and dermal IL-13 production in cGvHD-LPS^low^ mice, while their capacity for systemic IL-10 secretion was enhanced. Concomitantly, LPS^low^-macrophage infusion diminished CD69 expression on T and B cells and ICOS expression on CD4^+^ T cells. Current evidence suggests that dysregulated cytokine production occurs as a cascade during sequential macrophage and T-cell activation and is responsible for the clinical manifestation of the cGvHD (6). Also, IL-6 overproduction mediated by the translocation of microbiome-derived PAMPs perpetuates the inflammation in cGvHD (65), and its blockade alleviates the clinical severity (66). High amounts of TNF-*α* release following the conditioning regimen drive the initiating events of the GvHD and amplify the disease process once established through T-cell activation and apoptosis induction on target tissues (67). It has been shown that high levels of IFN-*γ* detected in a mixed lymphocyte culture in allogeneic bone marrow transplant recipient correlated with GVHD development in the corresponding patients (68). ICOS (CD278) is a costimulatory molecule expressed on activated T cells that favors Th17 and Th2 lymphocyte differentiation, and drives B cell interaction with germinal centers for antibody production (69). Its role in cGvHD is highly documented as recipients of *icos* ^(−/−)^ CD4^+^ T cells exhibited less GVHD morbidity (70) with a significant decrease in donor T follicular helper population, Th17 skewing, and lower antibody production (71).

Chemokines act on many aspects of the cGvHD pathophysiology including HSC homing and mobilization, T-cell activation, and immune cell recruitment to GvHD target organs (40). Adoptive transfer of LPS^low^-macrophages was accompanied with a drop in CCR4 and CCR10 expression on dermal CD4^+^ T cells as well as a decrease in CXCR3 expression on splenic T cells, along with a reduction in the skin *ccl17* and *ccl27 mRNA* content. The CCR10–CCL27 and CCR4–CCL17 chemokine axes play a critical role in CD4^+^ recruitment of T cells to inflamed skin (72, 73). Peripheral blood T cells from GvHD patients exhibit a high proportion of CD4^+^ CCR10^+^ T cells that disappear after GvHD resolution (74). CXCR3 is highly expressed on effector T cells upon activation and plays an important role in T-cell trafficking into inflamed tissues (75). Studies revealed a central role of CXCR3 in the pathogenesis of dermal damage during cGVHD (76), and its blockade inhibited allo-reactive CD8^+^ T cell and alleviated GvHD in mice (77). Here, the impairment of these chemokine axes may explain the attenuated skin damage and fibrosis observed in LPS^low^-cGvHD mice. After transplantation, naïve allogeneic CD4^+^ T cells were primed in the lymph nodes, proliferated and activated CD8^+^ T and B cells. This process induced effector T-cell populations and a progressive shift towards a predominant memory T-cell phenotype which was responsible for persistent host tissue injury (35, 78). CCR5 expression is essential to the recruitment of memory and effector T cells to the inflamed tissue (79), and this receptor has been linked to the pathogenesis of liver damage in GvHD (80). The blockade of CCR5 has also been described to inhibit leukocyte trafficking and reduce mucosal inflammation in murine colitis (81). Our findings indicate that the attenuation of the allo-immune response intensity and the lack of CCR5 expression on T cells provoked by LPS^low^-macrophages blocked the aberrant differentiation into memory phenotype and prevented cellular infiltrates into the liver and the intestine. Reduced levels of serum ALT as well as reduction in *mpo*, *il22*, and *il17* expression in the gut of LPS^low^-cGvHD mice, compared to the cGvHD controls, reflect the attenuated organ damage associated with the disease.

In sclerodermatous GvHD, visceral fibrosis is a hallmark of the advanced stage of disease and responsible for the devastating clinical outcome (82). Expression of *α-sma* and *col1a*, indicative of the activation of extracellular matrix secreting myofibroblasts, as well as the pro-fibrotic cytokines *il13* and *tgfb1* was notably reduced upon infusion of LPS^low^-macrophages. We have previously shown that the *in vivo* LPS^low^ training and adoptive transfer of LPS^low^-macrophages were effective in alleviating fibrotic manifestations in the skin and lungs in a murine model of systemic sclerosis (23). Fibrosis is a common pathological consequence of chronic inflammation and alteration of the immune response regulation (83). Here, the ability of LPS^low^-macrophages to dampen the inflammatory process by downregulating the T and B cell activation and reorganizing the networks of cytokines and chemokines counterbalanced the disproportionate immune response and prevented excessive fibrotic pathway activation and tissue damage in target organs.

Despite the differences in clinical phenotype and time of onset, acute and chronic murine GvHD share multiple immunopathological characteristics in which initial inflammation that drives and amplifies the allogeneic response remains the main actor (84). We showed that aGvHD mice infused with LPS^low^-macrophages had a marked improvement in overall survival and displayed attenuated clinical features. Numerous studies have proposed cellular therapy to hamper GvHD. So far, infusions of regulatory T-cells (Tregs), mesenchymal stromal cells (MSCs), and the Myeloid-Derived Suppressor Cells (MDSCs) have been the most studied, with promising results (9, 85), while the exact mechanism by which these cells improve GvHD outcome remains incompletely elucidated; multiple immunosuppressive properties such as IL-10, indoleamine 2-3 dioxygenase, ectonucleotidase, and arginase-1 production, and inhibitory T cell co-receptor expression have been suggested as mechanisms to suppress T cell function (86–88). As in cGvHD, the important inflammatory milieu associated with aGvHD acts as a trigger for the infused trained macrophages. In turn, those cells react with either an overproduction of inflammatory cytokines (BCG-macrophages) or an IL-10 release (LPS^low^-macrophages) that shuts down the inflammatory process. As GvHD is characterized by a very early overproduction of inflammatory cytokines and a short median survival, infusion of BCG-macrophages failed to mount an important synergistic effect that could translate clinically, while LPS^low^-macrophages treatment profoundly ameliorated the two clinical forms of the disease.

Successful treatment of GvHD is limited due to possible loss of the graft-*versus*-leukemia effect that leads to an increased relapse rate of a malignant hematological disease (89). The relapse usually results from immunosuppressive drugs that target host T cells and weaken their ability to recognize and control residual tumoral cells. In our A20 mice model of B lymphoma, infusion of LPS^low^-macrophages did not aggravate mortality rate nor accelerated tumor growth. Tumor growth is typically associated with the establishment of an immunosuppressive milieu that favors tumoral escape (90). Bascuas et al. reported a drop of a large array of chemokines and cytokines responsible for T-cell recruitment and activation within the tumor microenvironment using the A20 lymphoma cell murine model (91). Also, it has been shown that A20 tumor cells show altered expression of the costimulatory CD80 and are a potent source of IL-10 secretion, suggesting an important immunosuppressive status that accompanies A20 cell establishment (92). The lack of an inflammatory trigger in the context of a significant immunosuppression driven by the A20 cells may explain the absence of an added effect of the LPS^low^-macrophages to the tumoral growth. Notably, we observed that BCG-macrophage infusion attenuated tumor growth and was associated with an upregulation of the M1 macrophages associated genes including *ifng*, *il6*, and *inos* as well as with T cell activation and pro-inflammatory cytokine secretion. We and others have reported that BCG-trained macrophages acquire an M1-like phenotype with an enhanced capacity for TNF-*α* and IL-6 secretion and an overexpression of pattern recognition and co-stimulatory receptors (21, 23, 93). Macrophages are the most abundant cells within the tumor stroma, displaying a high plasticity with either beneficial or deleterious effects on tumor lesions (94). A shift toward the inflammatory M1 phenotype that orchestrates Th1 responses is classically associated with anti-tumoral effects and an improved prognosis for the underlying tumor (95). Although BCG immunotherapy is widely used in clinics to treat bladder cancer and has been tested successfully in malignant melanoma (96), the exact mechanism of its action is still debated (97). Our findings suggest that the anti-tumoral activity of BCG may act through macrophage training by conferring them a pro-inflammatory status that enhance their capacity to drive an efficient local immune response. Recent experiments have shown that the immunosuppressive status of myeloid cell can be successfully reversed by exposure to *β*-glucan, known to exert potent training effects on macrophages, resulting in a decreased frequency of MDSCs associated with lung carcinoma (98).

However, additional investigations are needed to assess the immunomodulatory impact of LPS^low^-macrophages on the separation of anti-leukemia effect from GvHD after BMT in an established GVL model.

This work is the first to highlight the immunomodulatory effects of trained macrophages on the course of two GvHD models, showing that cellular therapy may efficiently affect the immunological and fibrotic hallmark of this disease. Here, LPS^low^-macrophages strongly reduce the proliferation and the activation of allogeneic CD4^+^ T cells in an IL-10-dependent manner, early at the pathogenesis of the GvHD, slowing down the overwhelming inflammatory process that characterizes this disease. Survival time of injected macrophages has already been shown in previous studies to persist from 2 weeks to up to 1 year in some models and according to the macrophage tissue origin (99, 100). However, further experiments are needed to trace precisely the migration and the lifespan of the injected trained-macrophages. Importantly, our study opens the way to get further into the immune mechanisms of trained immunity by analyzing the effects of trained macrophages on the phenotype and function of T and B cells, and thus better understand how they shape the adaptive immune response. Furthermore, the novel immunotherapeutic approach addressed in this study is a promising approach to target either excessive immune response that drives graft rejection or defective immunity associated with malignant disease progression.

## Data Availability Statement

The original contributions presented in the study are included in the article/**Supplementary Material**. Further inquiries can be directed to the corresponding author.

## Ethics Statement

The animal study was reviewed and approved by the Ethics Committee from Paris Descartes University (N° CEEA34.CN.017.12).

## Author Contributions

Conceptualization, Methodology, MJ, FB and CN. Animal experiments MJ, BS, MT and CN. Investigation, MJ, BS, CN and CC. Formal analysis, MJ. Writing –Original Draft, MJ, CN and FB. Writing – Review, MJ, CN and LD. Editing, MJ and FB. Funding Acquisition, CN and FB. Supervision, CN and FB. All authors contributed to the article and approved the submitted version. 

## Funding

This work was supported by grants from University Paris Descartes INSERM.

## Conflict of Interest

The authors declare that the research was conducted in the absence of any commercial or financial relationships that could be construed as a potential conflict of interest.

## Publisher’s Note

All claims expressed in this article are solely those of the authors and do not necessarily represent those of their affiliated organizations, or those of the publisher, the editors and the reviewers. Any product that may be evaluated in this article, or claim that may be made by its manufacturer, is not guaranteed or endorsed by the publisher.

## Supplementary Material

Supplementary Figure 1 **(A)** Flow cytometric analysis of CD44 expression of CD4^+^ T cells in the MLR condition (*B10D2* CD4^+^ T cells with irradiated splenic *Balb/c* cells), *B10D2* CD4^+^ T cells incubated with anti CD3 and CD28 antibodies (Positive control), *B10D2* CD4^+^ T cells cultured alone and irradiated *Balb/c* splenocytes (Negative controls). Each bar represents the mean fluorescence intensity of CD44 expression ± SEM obtained from the 10-plicate of three independent experiments. **(B)** Proliferation index of CFSE-stained cells in the MLR condition (*B10D2* CD4^+^ T cells cultured with irradiated splenic *Balb/c* cells), *B10D2* CD4^+^ T cells incubated with anti CD3 and CD28 antibodies (Positive control), B10D2 CD4^+^ T cells cultured alone and irradiated Balb/c splenocytes (Negative controls). Each bar represents the mean proliferation index of ± SEM obtained from the 10-plicates of three independent experiments. **(C)** ELISA assessment of IL-2, IFN-*γ*, TNF-*α*, IL-6, and IL-10 cytokine concentrations in the supernatant of the different MLR control conditions. Bar graphs represent the mean (pg/ml) ± SEM of 10-plicate of three independent experiments. The ANOVA test with Bonferroni correction was used to detect significant differences between the groups. Statistics are shown between the MLR group and the different control conditions. NS, non-significant, *p ≤ 0.05; **p ≤ 0.01; ***p ≤ 0.001.

Supplementary Figure 2 **(A)** Frequency of naive and memory CD4^+^ T-cell subpopulations in the spleen of the syngeneic *versus* the cGvHD groups. Data represent the absolute count of naive (CD62L^High^ CD44^Low^) and memory (CD62L^Low^ and CD44^High^) CD4^+^ T cells among the total splenic CD4^+^ T-cell population ± SEM obtained from at least n = 5 biologically independent mice. **(B)** Flow cytometric measurement of the CD69 expression on splenic CD4^+^ and CD8^+^ T cells and B220^+^ B cells. **(C)** Flow cytometric analysis of ICOS expression on splenic CD4^+^ and CD8^+^ T cells. **(D)** Cytokine production in the supernatant of stimulated splenic cells from the syngeneic and the cGvHD groups. Levels of IFN-*γ*, TNF-*α*, IL-17, and IL-10 were assessed by ELISA and concentrations are expressed in pg/ml. Data represent the mean ± SEM from the duplicate of at least n = 5 biologically independent samples. **(E)** ELISA assessment of IL-13 production (pg/ml) by skin-derived immune cells from the syngeneic and the cGvHD mice. Each box represents mean ± SEM of triplicate obtained with cell culture from at least n = 5 biologically independent samples. The ANOVA test with Bonferroni correction was used to detect significant differences between the groups. NS, non-significant; *p ≤ 0.05; **p ≤ 0.01; ***p ≤ 0.001.

Supplementary Figure 3 **(A, B)** Histograms showing the absolute count of dermal CD4^+^ T and CD8^+^ T-cell populations. Cell suspensions were obtained from the lysis of dermal punches of tissue collected from the shaved back of each mouse. Statistics are shown between the cGvHD group and the cGvHD + trained macrophages groups. The ANOVA test with Bonferroni correction was used to detect significant differences between the groups. NS: non-significant; *p ≤ 0.05; **p ≤ 0.01; ***p ≤ 0.001. **(C)** Representative skin and lung sections of 5 μm stained with hematoxylin and eosin in the different groups. Overall, cGvHD mice have an increased dermis or inter-alveolar mononuclear cell infiltration compared to syngeneic group. Photographs were taken with a Nikon Eclipse 80i microscope. Original magnification ×40.

Supplementary Figure 4 **(A)** Flow cytometric gating strategy to detect dermal CD4^+^ and CD8^+^ T cells from *Balb/c* mice for further analysis of surface marker expression. CD4^+^ T cells were identified as CD3^+^ CD4^+^ double positive cells and CD8^+^ T cells were identified as CD3^+^ CD8^+^ double positive cells. **(B)** Flow cytometric gating strategy to detect splenic CD4^+^ and CD8^+^ T cells from *Balb/c* mice for further analysis of surface markers expression. CD4^+^ T cells were identified as CD3^+^ CD4^+^ double positive cells and CD8^+^ T cells were identified as CD3^+^ CD8^+^ double positive cells. B cells were identified as CD3^-^ B220^+^. **(C)** Flow cytometric gating strategy to identify splenic macrophages from *Balb/c* mice for further surface marker analysis. Total splenic cells were gated on CD11b^+^ and F4/80^+^ to identify macrophage population.
