# Supplementary figures and images for: LPSlow-Macrophages Alleviate the Outcome of Graft-Versus-Host Disease Without Aggravating Lymphoma Growth in Mice
Source: Front Immunol. 2021 Aug 3;12:670776. doi: 10.3389/fimmu.2021.670776 (PMC8369416; doi:10.3389/fimmu.2021.670776)

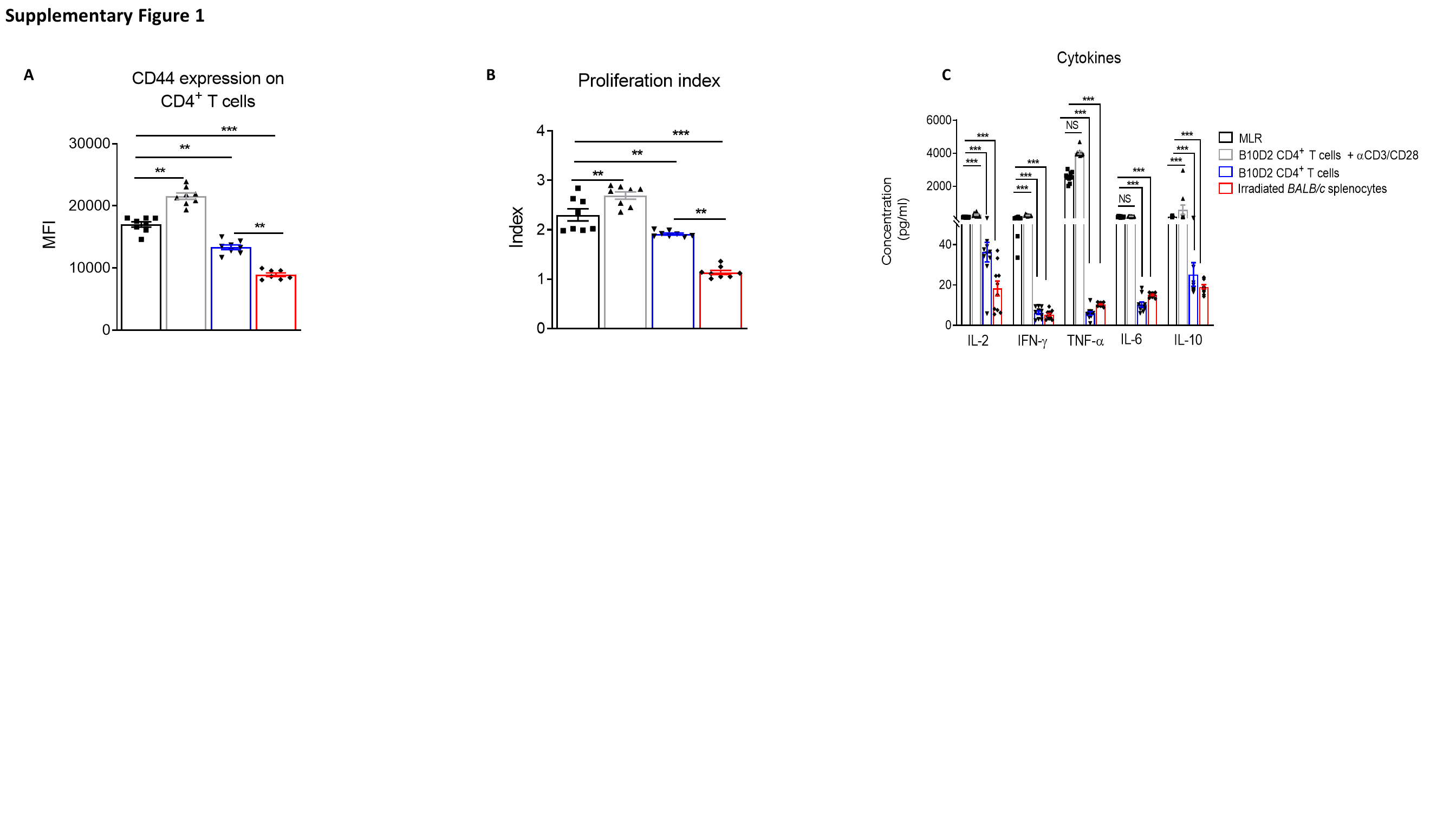

Supplement: Supplementary Figure 1 — (A) Flow cytometric analysis of CD44 expression of CD4+ T cells in the MLR condition (B10D2 CD4+ T cells with irradiated splenic Balb/c cells), B10D2 CD4+ T cells incubated with anti CD3 and CD28 antibodies (Positive control), B10D2 CD4+ T cells cultured alone and irradiated Balb/c splenocytes (Negative controls). Each bar represents the mean fluorescence intensity of CD44 expression ± SEM obtained from the 10-plicate of three independent experiments. (B) Proliferation index of CFSE-stained cells in the MLR condition (B10D2 CD4+ T cells cultured with irradiated splenic Balb/c cells), B10D2 CD4+ T cells incubated with anti CD3 and CD28 antibodies (Positive control), B10D2 CD4+ T cells cultured alone and irradiated Balb/c splenocytes (Negative controls). Each bar represents the mean proliferation index of ± SEM obtained from the 10-plicates of three independent experiments. (C) ELISA assessment of IL-2, IFN-γ, TNF-α, IL-6, and IL-10 cytokine concentrations in the supernatant of the different MLR control conditions. Bar graphs represent the mean (pg/ml) ± SEM of 10-plicate of three independent experiments. The ANOVA test with Bonferroni correction was used to detect significant differences between the groups. Statistics are shown between the MLR group and the different control conditions. NS, non-significant, *p ≤ 0.05; **p ≤ 0.01; ***p ≤ 0.001. [file Image_1.tif]

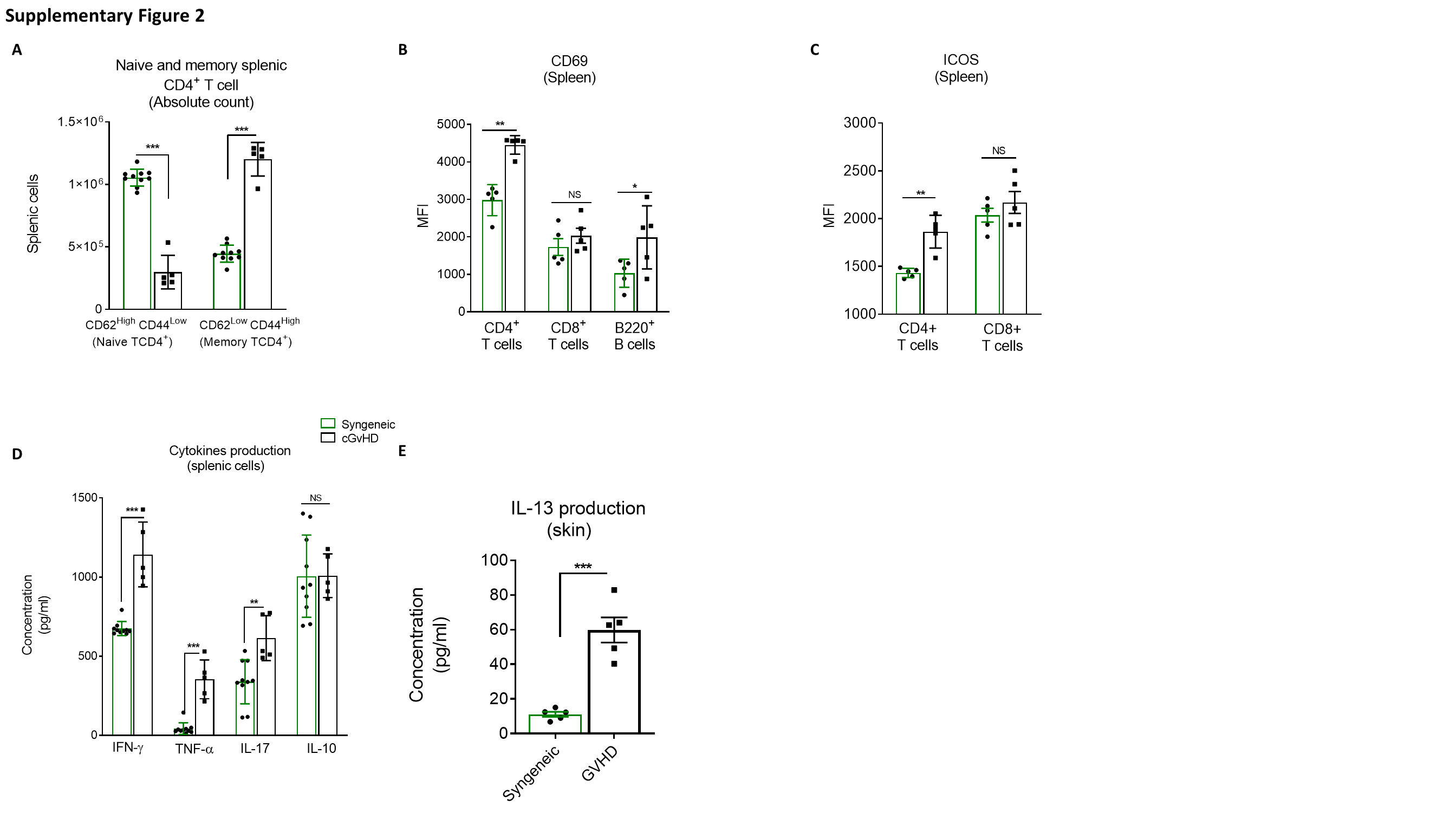

Supplement: Supplementary Figure 2 — (A) Frequency of naive and memory CD4+ T-cell subpopulations in the spleen of the syngeneic versus the cGvHD groups. Data represent the absolute count of naive (CD62LHigh CD44Low) and memory (CD62LLow and CD44High) CD4+ T cells among the total splenic CD4+ T-cell population ± SEM obtained from at least n = 5 biologically independent mice. (B) Flow cytometric measurement of the CD69 expression on splenic CD4+ and CD8+ T cells and B220+ B cells. (C) Flow cytometric analysis of ICOS expression on splenic CD4+ and CD8+ T cells. (D) Cytokine production in the supernatant of stimulated splenic cells from the syngeneic and the cGvHD groups. Levels of IFN-γ, TNF-α, IL-17, and IL-10 were assessed by ELISA and concentrations are expressed in pg/ml. Data represent the mean ± SEM from the duplicate of at least n = 5 biologically independent samples. (E) ELISA assessment of IL-13 production (pg/ml) by skin-derived immune cells from the syngeneic and the cGvHD mice. Each box represents mean ± SEM of triplicate obtained with cell culture from at least n = 5 biologically independent samples. The ANOVA test with Bonferroni correction was used to detect significant differences between the groups. NS, non-significant; *p ≤ 0.05; **p ≤ 0.01; ***p ≤ 0.001. [file Image_2.tif]

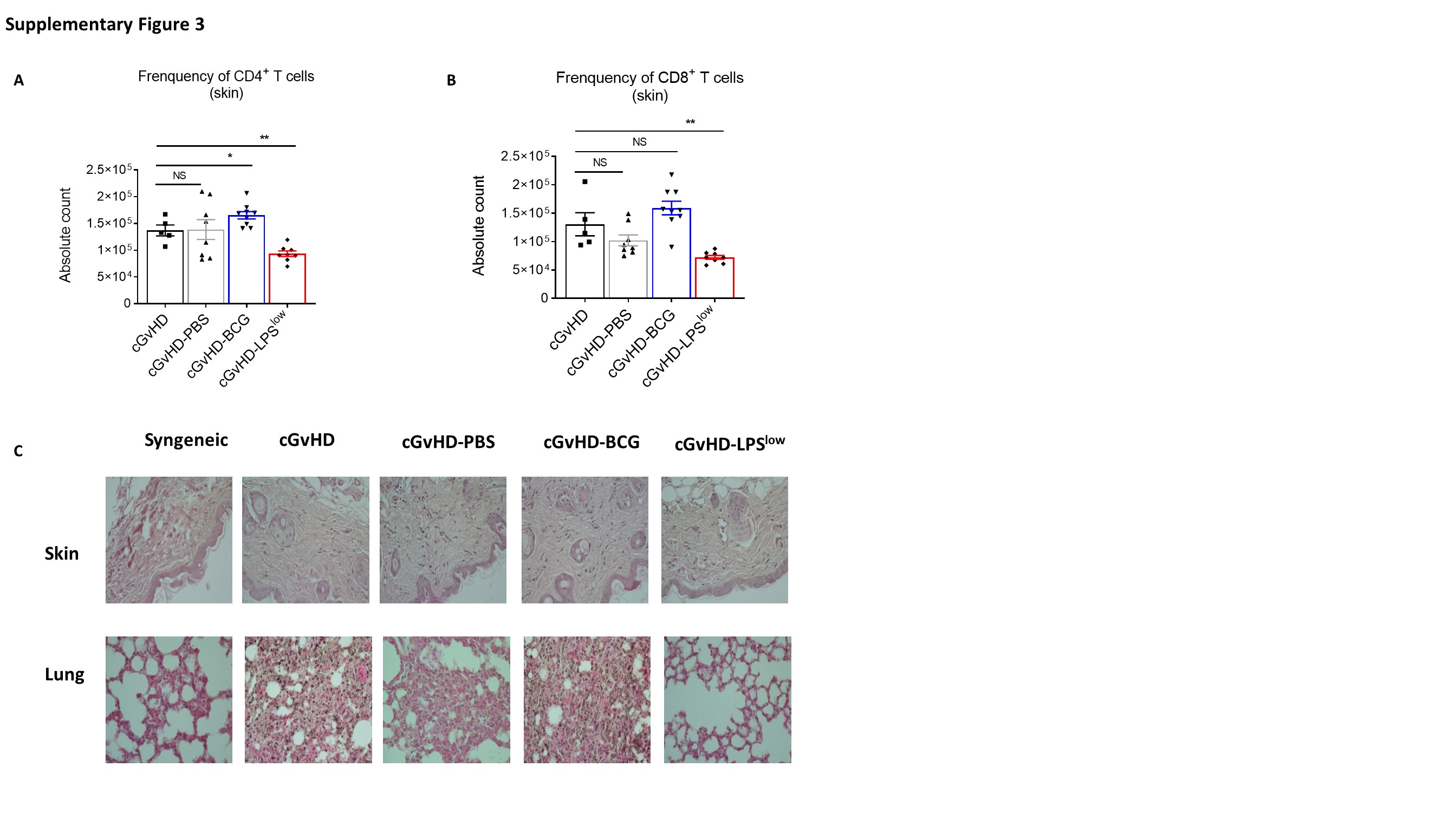

Supplement: Supplementary Figure 3 — (A, B) Histograms showing the absolute count of dermal CD4+ T and CD8+ T-cell populations. Cell suspensions were obtained from the lysis of dermal punches of tissue collected from the shaved back of each mouse. Statistics are shown between the cGvHD group and the cGvHD + trained macrophages groups. The ANOVA test with Bonferroni correction was used to detect significant differences between the groups. NS: non-significant; *p ≤ 0.05; **p ≤ 0.01; ***p ≤ 0.001. (C) Representative skin and lung sections of 5 μm stained with hematoxylin and eosin in the different groups. Overall, cGvHD mice have an increased dermis or inter-alveolar mononuclear cell infiltration compared to syngeneic group. Photographs were taken with a Nikon Eclipse 80i microscope. Original magnification ×40. [file Image_3.tif]

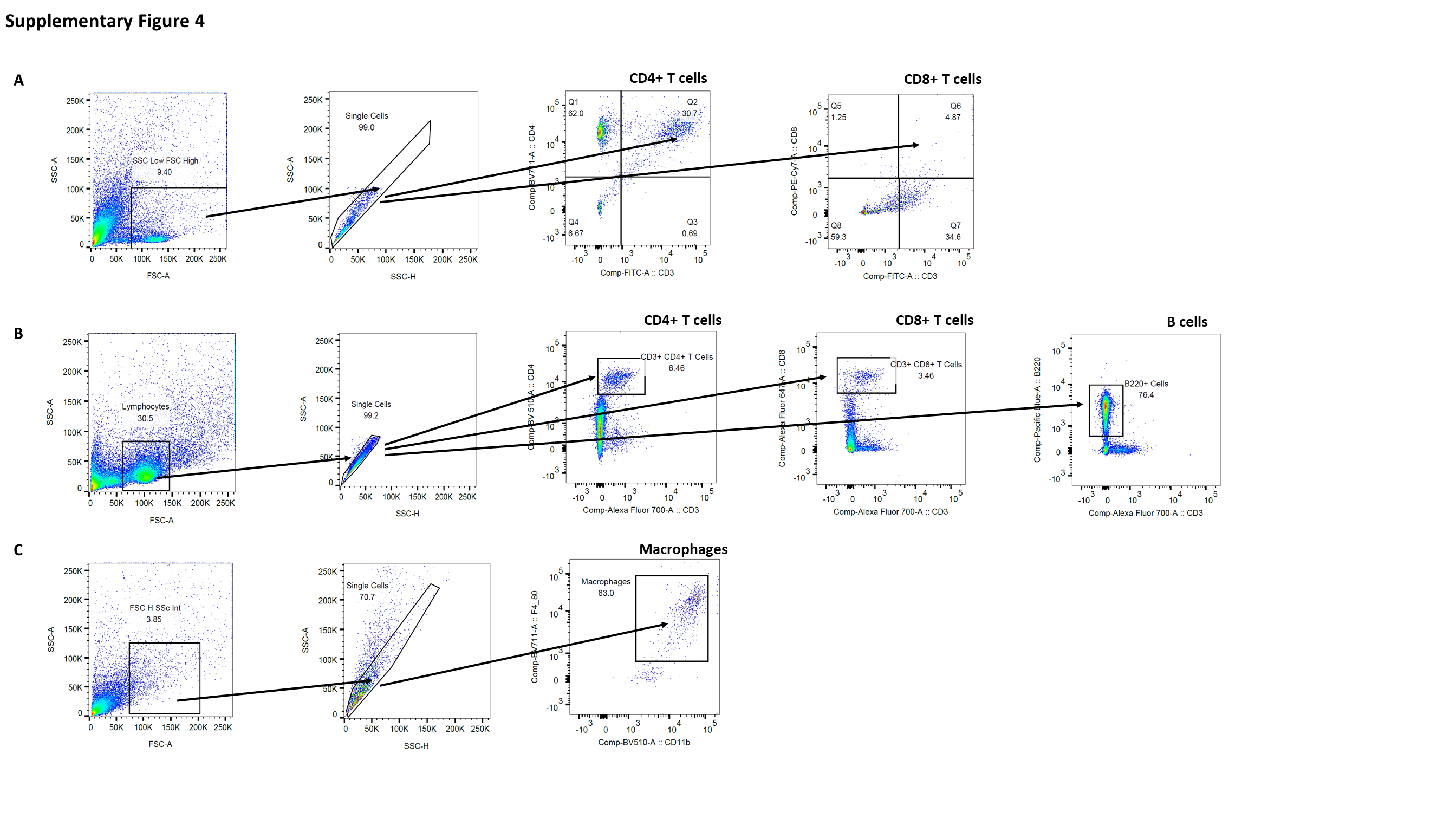

Supplement: Supplementary Figure 4 — (A) Flow cytometric gating strategy to detect dermal CD4+ and CD8+ T cells from Balb/c mice for further analysis of surface marker expression. CD4+ T cells were identified as CD3+ CD4+ double positive cells and CD8+ T cells were identified as CD3+ CD8+ double positive cells. (B) Flow cytometric gating strategy to detect splenic CD4+ and CD8+ T cells from Balb/c mice for further analysis of surface markers expression. CD4+ T cells were identified as CD3+ CD4+ double positive cells and CD8+ T cells were identified as CD3+ CD8+ double positive cells. B cells were identified as CD3- B220+. (C) Flow cytometric gating strategy to identify splenic macrophages from Balb/c mice for further surface marker analysis. Total splenic cells were gated on CD11b+ and F4/80+ to identify macrophage population. [file Image_4.tif]
