# Supplementary material for: LPSlow-Macrophages Alleviate the Outcome of Graft-Versus-Host Disease Without Aggravating Lymphoma Growth in Mice
Source: Front Immunol. 2021 Aug 3;12:670776. doi: 10.3389/fimmu.2021.670776 (PMC8369416; doi:10.3389/fimmu.2021.670776)
Supplement: Supplementary file 5 [file Table_1.pdf]

Supplementary Table 1 : List of primers sequences used for real time quantitative PCR

Murine primers used for RT-qPCR analysis for tissues and cells		
Gene	Primer sequence 5'-3' (F)	Primer sequence 5'-3' (R)
β-actin	ACCACCATGTACCCAGGCATT	CCACACAGAGTACTTGCGCTCA
col1a1	TGTTCGTGGTTCTCAGGGTAG	TTGTCGTAGCAGGGTTCTTTC
asma	GCATCCACGAAACCACCTA	CACGAGTAACAAATCAAAGC
tgfb1	CACCGGAGAGCCCTGGATA	TGTACAGCTGCCGCACACA
mpo	TGAATCCTCGATGGAATGGG	ACGGAAAGCGTTGGTGAAGA
fizz1	TATGAACAGATGGGCCTCCT	CCACTCTGGATCTCCCAAGA
inos	GCCCAGCCAGGTACAGAG	CCTTGGTGCAGAAACCCTTA
il4	AACGAGGTCACAGGAGAAGG	TCTGCAGCTCCATGAGAACA
il6	GAGGATACCACTCCCAACAGACC	AAGTGCATCATCGTTGTTCATACA
il10	TGAGGCGCTGTCGTCATCGATTTCTCCC	ACCTGCTCCACTGCCTTGCT
il13	GCAGCATGGTATGGAGTGTG	TGGCGAAACAGTTGCTTTGT
il17	TTTAACTCCCTTGGCGCAAAA	CTTTCCCTCCGCATTGACAC
il22	ATGAGTTTTTCCCTTATGGGGAC	GCTGGAAGTTGGACACCTCAA
ccl17	CAGGAAGTTGGTGAGCTGGT	GGGTCTGCACAGATGAGCTT
ccl27	AGTTTAGGCTGTGCCGACC	GCCCCAGTAGATCTCCTCCT
ccn2	GGGCCTCTTCTGCGATTTC	ATCCAGGCAAGTGCATT
sox9	GAGCCGGATCTGAAGAAGGA	GCTTGACGTGGCTTGTTC
krt19	GGGGGTTCAGTACGCATTGG	GAGGACGAGGTCACGAAGC
cd40l	CCTTGCTGAACTGTGAGGAGA	CTTCGCTTACAACGTGTGCT
cxcl2	AAGTTTGCCTTGACCCTGAA	AGGCACATCAGGTACGATCC
ccl2	TTAAAAACCTGGATCGGAACCAA	GCATTAGCTTCAGATTTACGGGT
arnt2	TTATCACGTTTGTGGACCCCA	GTTGGTGCAGGTGACGTACT
