# Supplementary material for: LPSlow-Macrophages Alleviate the Outcome of Graft-Versus-Host Disease Without Aggravating Lymphoma Growth in Mice
Source: Front Immunol. 2021 Aug 3;12:670776. doi: 10.3389/fimmu.2021.670776 (PMC8369416; doi:10.3389/fimmu.2021.670776)
Supplement: Supplementary file 6 [file Table_2.pdf]

Supplementary Table 2 : List of antibodies used for the Western Blot experiment

Antibodies	Dilution	Source	Identifier
Goat anti-Rabbit IgG (H+L) Secondary Antibody, HRP	1/10000	Thermo Fisher Scientific	#A27036
STAT3 IgG	1/200	Santa Cruz	#sc-8019
pSTAT3 IgG2b	1/200	Santa Cruz	#sc-8059
IL-10 IgG2b	1/1000	Santa Cruz	#sc-365858
Mouse anti-β-Actin−Peroxidase antibody	1/10000	Sigma Aldrich	#A3854
Cdk4	1/500	Santa Cruz	#sc-260
Cyclin A	1/500	Santa Cruz	#sc271682
ZAP-70	1/2000	Cell Signaling	#3165
pZAP-70	1/1000	Cell Signaling	#2717
Goat anti-Mouse IgG (H+L) Secondary Antibody, HRP	1/10000	Thermo Fisher Scientific	#A28177
